# Inhibition of colony stimulating factor 1 receptor corrects maternal inflammation-induced microglial and synaptic dysfunction and behavioral abnormalities

**DOI:** 10.1038/s41380-020-0671-2

**Published:** 2020-02-18

**Authors:** Seiko Ikezu, Hana Yeh, Jean-Christophe Delpech, Maya E. Woodbury, Alicia A. Van Enoo, Zhi Ruan, Sudhir Sivakumaran, Yang You, Carl Holland, Teresa Guillamon-Vivancos, Asuka Yoshii-Kitahara, Mina B. Botros, Charlotte Madore, Pin-Hao Chao, Ankita Desani, Solaiappan Manimaran, Srinidhi Venkatesan Kalavai, W. Evan Johnson, Oleg Butovsky, Maria Medalla, Jennifer I. Luebke, Tsuneya Ikezu

**Affiliations:** 1 Departments of Pharmacology and Experimental Therapeutics, Boston University School of Medicine, Boston, MA, USA; 2 Graduate Program in Neuroscience, Boston University, Boston, MA, USA; 3 Anatomy and Neurobiology, Boston University School of Medicine, Boston, MA, USA; 4 Ann Romney Center for Neurologic Diseases, Department of Neurology and Evergrande Center for Immunologic Diseases, Brigham and Women’s Hospital, Harvard Medical School, Boston, MA, USA; 5 Computational Biomedicine, Boston University School of Medicine, Boston, MA, USA; 6 Center for Systems Neuroscience, Boston University, Boston, MA, USA; 7 Department of Neurology and Alzheimer’s Disease Center, Boston University School of Medicine, Boston, MA, USA

## Abstract

Maternal immune activation (MIA) disrupts the central innate immune system during a critical neurodevelopmental period. Microglia are primary innate immune cells in the brain although their direct influence on the MIA phenotype is largely unknown. Here we show that MIA alters microglial gene expression with upregulation of cellular protrusion/neuritogenic pathways, concurrently causing repetitive behavior, social deficits, and synaptic dysfunction to layer V intrinsically bursting pyramidal neurons in the prefrontal cortex of mice. MIA increases plastic dendritic spines of the intrinsically bursting neurons and their interaction with hyper-ramified microglia. Treating MIA offspring by colony stimulating factor 1 receptor inhibitors induces depletion and repopulation of microglia, and corrects protein expression of the newly identified MIA associated neuritogenic molecules in microglia, which coalesces with correction of MIA-associated synaptic, neurophysiological, and behavioral abnormalities. Our study demonstrates that maternal immune insults perturb microglial phenotypes and influence neuronal functions throughout adulthood, and reveals a potent effect of colony stimulating factor 1 receptor inhibitors on the correction of MIA-associated microglial, synaptic, and neurobehavioral dysfunctions.

## Introduction

Viral or bacterial infection during the first trimester of pregnancy has been associated with a higher incidence of neurodevelopmental disorders [1, 2]. Animal models of maternal immune activation (MIA) have been widely studied to understand their underlying pathogenesis. MIA is commonly induced in pregnant rodents by the viral mimetic Toll-like receptor 3 agonist polyinosinic:polycytidylic acid [poly(I:C)] [3]. MIA offspring exhibit long-lasting behavioral abnormalities, such as increased repetitive behavior as well as impaired social interaction and communication [3]. Interestingly, MIA offspring also display altered cytokines in the brain including the frontal cortex and hippocampus from birth through adulthood [4], suggesting that chronic neuroimmune modulation may play a role in mediating the deleterious effect of prenatal immune challenge on neurodevelopment. Microglia, the primary innate immune cells in the central nervous system, could mediate the MIA phenotype. They are the first immune responders in the brain and support synaptic maturation and circuit formation during critical developmental periods [5–7]. Upon exposure to immune stimuli from infection or injury, microglia are known to adapt their activities to resolve pathological events and maintain brain homeostasis. Impaired restoration of microglial homeostatic phenotype can lead to long-lasting modifications of brain function [8]. Indeed, microglia retain an MIA-induced phenotype after the initial immune challenge, as manifested by altered microglial morphology, gene, and protein expression [8–13]. Microglial transcriptome analyses revealed that poly(I:C) injected MIA offspring showed shifting of early microglia to more developmental stage at P1 [10] or reduced expression of sensome molecules related to phagocytosis at P120, which was prevented by the administration of minocycline, an anti-inflammatory drug [13]. Those results indicate that early immune insults may change the course of microglial development and phenotype, thereby dysregulate their physiological function to support neuronal development and plasticity. However, the exact mechanisms of how perturbed microglia interfere with neuronal circuits and induce behavioral abnormalities in adult MIA offspring remain unclear. To elucidate the microglial contribution to MIA phenotype at adulthood, we examined the effect of microglial depletion. Microglial depletion has been accomplished through genetic methods or by injection of clodronate liposomes or pharmacological inhibitors of colony stimulating factor-1 receptor (CSF1R) [6, 14, 15]. CSF1R is exclusively expressed by microglia in the brain, and its inhibition efficiently depletes microglia with minimal effects on neurons, astrocytes, or peripheral immune populations [15]. Recent studies suggest that depleting microglia by CSF1R inhibition induce local repopulation from selfrenewed microglia [16, 17] and those replenished microglia may reverse aging and inflammation-induced neuropathology with a healthier population [18]. Here we report that administration of CSF1R inhibitor followed by microglial repopulation reversed MIA-induced abnormal behavior in the offspring. CSF1R inhibitor treatment corrected microglial phenotypic changes and aberrant interactions with neurons, accompanied by normalized synaptic properties and spine morphologies.

## Results

### Microglial repopulation corrects repetitive behavior and social deficits in MIA offspring

**Fig. 1:**
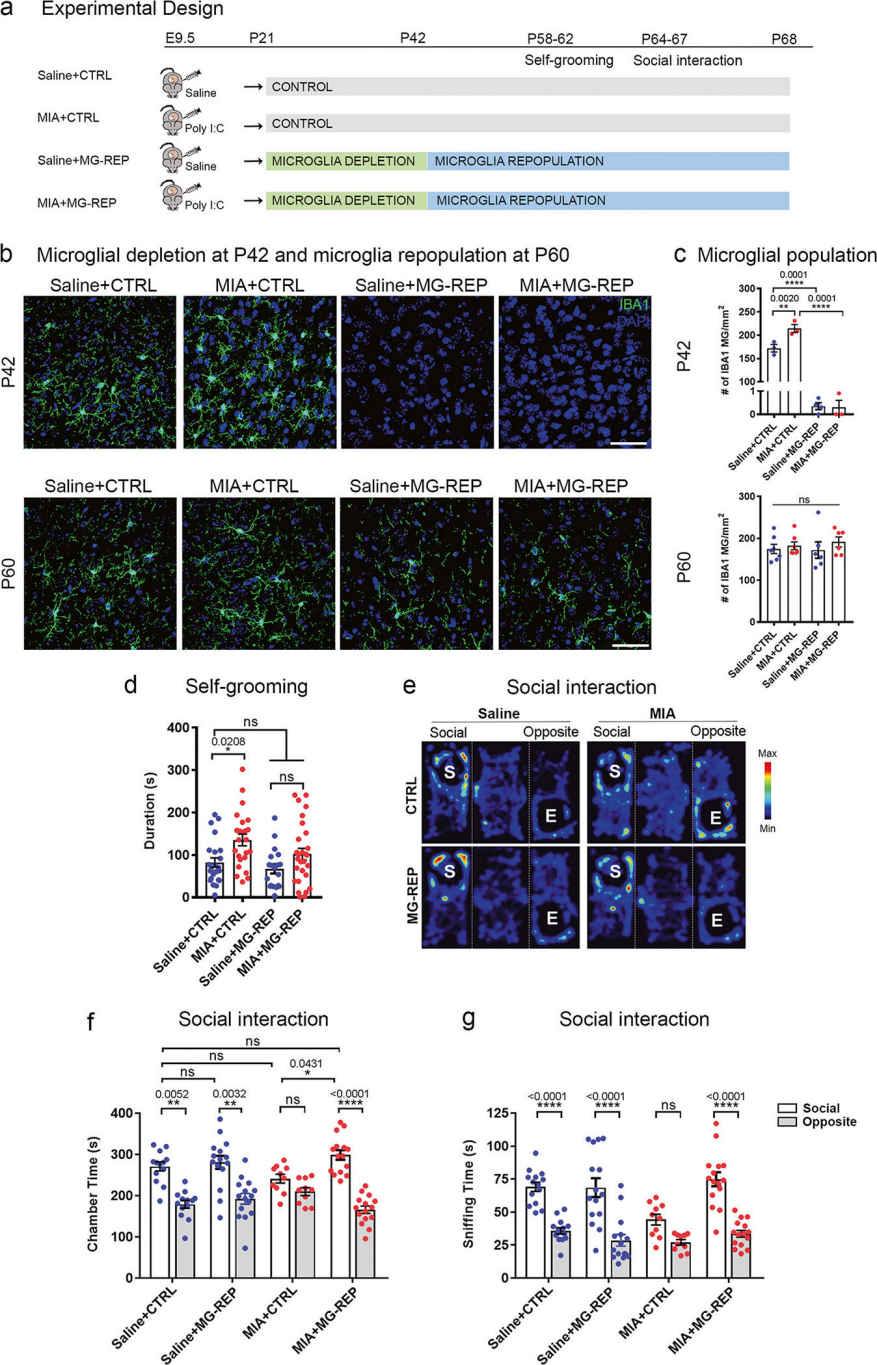
Microglia depletion and repopulation corrects repetitive behavior and social deficits in MIA offspring. **a** Timeline of drug treatment and behavioral testing. **b** Immunostaining of Saline + CTRL, MIA + CTRL, Saline + MG-REP and MIA + MG-REP animals at P42, after 3 weeks of PLX, and P60 after 18 days for repopulation, in the medial prefrontal cortex (mPFC), with antibodies against IBA1 (green) and DAPI (blue). Scale bar = 10 μm. **c** Quantification of IBA1^+^ cells in the mPFC showing complete microglial depletion by CSF1R inhibitor treatment and full recovery of microglia at P60. *n* = (3/2, 3/2, 4/2, 3/2) for male mice/litters per group P42 and *n* = (7/5, 7/5, 6/5, 6/3) male mice/litters per group for p60 (Saline + CTRL, MIA + CTRL, Saline + PLX and MIA + PLX). There was a main effect of prenatal treatment *F*(1,9) = 16.81, *p* = 0.0027 as well as main effect of drug treatment *F*(1,9) = 1486 *p* < 0.0001 and interaction effect *F*(1,9) = 16.31, *p* = 0.0029, at P42. **d** MIA male offspring show repetitive behavior deficits as shown in increased time spent grooming. Prenatal treatment effect, *F*(1,90) = 11.62, *P* = 0.0010, Drug effect, *F*(1,90) = 3.525, *p* = 0.0637, Interaction effect, *F*(1,90) = 0.5208, *p* = 0.4724, *n* = (23/9, 24/9, 19/11, 28/7) male mice/litters for (Saline + CTRL, MIA + CTRL, Saline + MG-REP, MIA + MG-REP). **e–g** Corrected social behavior in MIA male offspring by MG-REP: **e** Representative heatmaps of Three Chamber Social Interaction Test, “S”: tube containing social mouse, “E”: empty tube. **f** Difference in duration of time spent in “social” versus “opposite” chambers in saline offspring (left) and MIA offspring (right). Prenatal treatment effect, *F* (1,49) = 0.1947, *P* = 0.6610, Drug effect, *F*(1,49) = 5.289, *p* = 0.0258, chamber effect, *F*(1,49) = 57.20, *p* < 0.0001, Interaction effect, *F*(1,49) = 5.296, *p* = 0.0257. **g** Difference in duration of time spent sniffing the “social” versus empty “opposite” cylinders in saline offspring (left) and MIA offspring (right). Prenatal treatment effect, *F* (1,50) = 3.632, *P* = 0.0624, Drug effect, *F*(1,50) = 6.354, *p* = 0.0149, chamber effect, *F*(1,50) = 87.69, *p* < 0.0001, Interaction effect, *F*(1,50) = 1.544, *p* = 0.2198. *n* = (15/5, 15/9, 12/5, 15/4) male mice/ litters for (Saline + CTRL, Saline + MG-REP, MIA + CTRL, MIA + MG-REP), *p < 0.05; **p < 0.01; ****p < 0.0001, ns denotes no significance; determined by 3-way ANOVA (alpha = 0.05) and Sidak post-hoc. Graphs indicate mean ± s.e.m.

First, we asked whether repopulating microglia following the depletion by a CSF1R inhibitor would correct abnormal behaviors in MIA offspring [14, 15]. MIA was induced by the injection to pregnant C57BL6/J mice of 20 mg/kg poly (I:C) (“MIA”) or saline (“Saline”) on embryonic day 9.5 (E9.5), which corresponds to the beginning of microglial migration from the yolk sac into the brain in mice [19] and to the first trimester in humans [20]. The control chow treatment groups (“CTRL”) were fed with a control chow from P21–68. The microglial repopulation treatment groups (“MG-REP”) were fed chow containing a CSF1R inhibitor (PLX5622, “PLX”) from P21–42, followed by control chow from P42–68 (Fig. 1a). In order to determine the effect of PLX on microglia, we evaluated microglial number of male offspring by immunohistochemistry against phagocyte marker ionized calcium biding adapter molecule 1 (IBA1) in the medial prefrontal cortex (mPFC), the area implicated in aberrant neuronal circuitry and synchrony in MIA [21] (Fig. 1b). After 3 weeks of CTRL or PLX diet at P42, we found complete microglial depletion by PLX treatment in both Saline (99.8%) and MIA (99.9%) offspring and their recovery at P60, 18 days after PLX treatment (Fig. 1b, c). MIA increased microglial density in the mPFC compared with CTRL offspring at P42, which was not observed at P60 (Fig. 1c), suggesting the transient increase of microglia by MIA, which is in line with the previous studies [22, 23].

MIA offspring display abnormal vocal communication, social interaction and repetitive behaviors [3, 24]. We first examined their repetitive behaviors with male offspring by the self-grooming test at P58–62 [3]. We found significant increase in the total self-grooming time between Saline + CTRL and MIA + CTRL, which was diminished by PLX treatment. There was no siginifcant difference in duration time per bout in any of the groups (Fig. 1d and Supplementary Fig. 1a).

Next, we performed the three-chamber test with male offspring in adulthood at P64–67 to assess MIA effect on sociability and measured the total time spent in the social versus opposite empty chamber (Fig. 1e–g). Saline + CTRL offspring showed significant social preference, whereas MIA + CTRL offspring displayed a reduction of social preference. Strikingly, MIA + MG-REP offspring spent significantly more time in the social chamber than in the opposite chamber, and significantly more time in the social chamber compared with MIA + CTRL offspring, indicating that the reduction of social preference observed in MIA + CTRL offspring was corrected by PLX treatment (Fig. 1f).

We found consistent results by measuring sniffing time with the social mouse cylinder (S) compared with the empty cylinder (E), confirming the treatment effect of PLX on social preference (Fig. 1g). There was no significant difference in the center time and overall distance traveled between the groups (Supplementary Fig. 1b, c). In addition, we recorded ultrasonic vocalizations (USV) with male offspring to assess changes in communication by MIA. However, the adult social deficit may occur through a nonvocal mechanism, since we did not find any significant difference in the total number of calls among the four groups at this age (Supplementary Fig. 1d). Taken together, these results demonstrate that MIA offspring show enhanced repetitive behaviors, reduced social preference, and that PLX treatment may rescue MIA-induced behavioral abnormalities.

### Microglial transcriptome reveals novel MIA and repopulation modules and their overlap with ASD risk genes

Microglia are not only immune surveyors, but also play critical roles in synapse formation [6] and neuronal connectivity [6]. They maintain homeostatic and immunological functions through control of sensome and immune signaling, which are regulated by transcriptional networks [10].

**Fig. 2:**
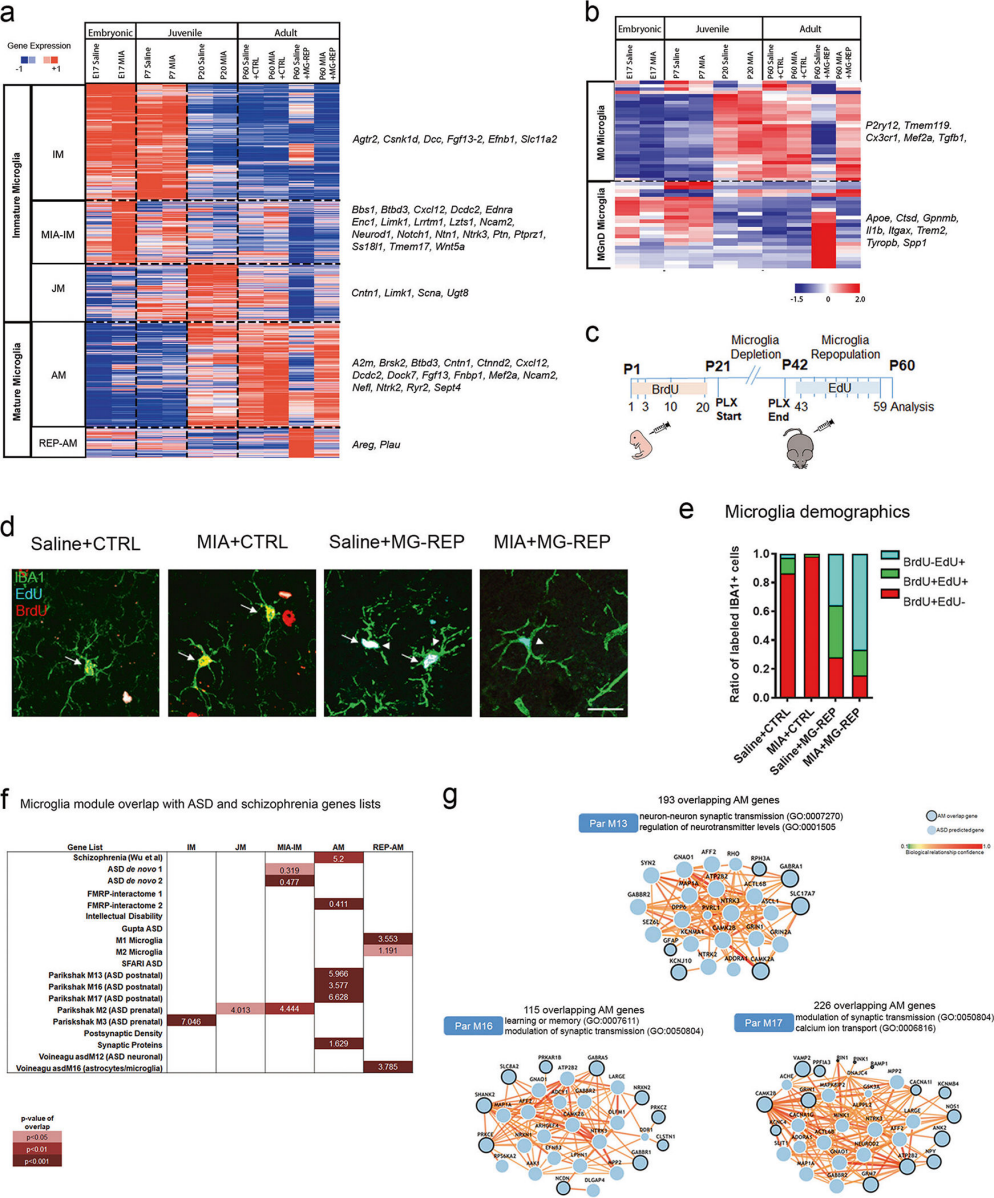
Microglial transcriptome reveals novel MIA and repopulation modules and overlap of MIA microglial genes with ASD gene network. **a** Heatmap (*z*-scores) of differentially expressed genes in acutely isolated microglia in five modules: immature microglia (IM), MIA immature microglia (MIA-IM), juvenile microglia (JM), adult microglia (AM), repopulated adult microglia (REP-AM). *n* = 3 microglia samples per group, one sample consists of one litter for E17, three pooled brains for P7, and one brain for P20 and P60 (male and female). **b** Heatmap (*z*-scores) of differentially expressed genes in acutely isolated microglia in M0 (homeostatic microglia) and DAM/MGnD-specific genes (E17-P60: male and female microglia). Fate mapping analysis of repopulating microglia. **c** Schematic diagram of BrdU and EdU injections from P1 to P59. BrdU and EdU were injected in a total of five (P1, 2, 3, 10, and 20) and eight times (every other day from P43–59), respectively. **d** Representative images of BrdU (red), EdU (cyan), and IBA1 (green) microglia for Saline + CTRL,MIA + CTRL, Saline + MG-REP and MIA + MG-REP. Arrow shows BrdU^+^IBA1^+^ original microglia. Arrowhead shows EdU^+^IBA1^+^ repopulated microglia. Scale Bar = 20 μm. e Demographics of all groups in cortex: BrdU^+^EdU^−^ originally dividing microglia (red), BrdU^+^EdU^+^ repopulated originally dividing microglia (green), BrdU^−^EdU^+^ repopulated originally non-dividing microglia (blue). (19/4/2, 12/3/2, 18/4/2, 20/4/2) sections/female mice/litters for Saline + CTRL, MIA + CTRL, Saline + MG-REP and MIA + MG-REP. **f** Microglia module overlap analysis with human neurodevelopmental disorder-associated gene modules. Color scale denotes *p* value of hypergeometric distribution analyses. Numbers on table denote percent overlapped genes out of total number of genes in the modules with significant overlap (*p* < 0.05). **g** Network generation of overlapping human ASD modules (ParM13, M16, and M17) and AM microglia identify common genes underlying AM-associated molecular networks and highlight the contribution of microglia to the pathogenesis of human neurodevelopmental disorders. The overall connectedness of the gene list for each module was greater than all 100,000 permuted gene lists, yielding *p* < 0.00001.

To determine the transcriptomic changes underlying the MIA microglia phenotype and their reversal by PLX, we performed RNA-sequencing of magnetic CD11B beadsisolated microglia from the whole brain in Saline and MIA male and female offspring at E17, P7, P20, and P60, as well as from MG-REP male and female offspring at P60. In order to verify the purity of RNA-seq across ages and conditions, we assessed expression of cell-type specific markers (derived from Barres dataset of mouse neurons, astrocytes, oligodendrocytes, endothelial cells, and microglia) [25], which demonstrate an average of 98% purity, in agreement with the purity calculated from Zhang et al. (97.8%) [25] and a FACS purified microglia RNA-seq database (98%) [26]. Since RNA-sequencing samples were from both genders, we evaluated sex as possible confounding effects. Based on this analysis and our balanced experimental design, we concluded that there was no evidence that this variable modified the effect of treatment or any of our downstream conclusions. *K*-means clustering (*k* = 5) of 14,225 microglial genes from RNA-seq data revealed distinct transcriptional signatures of immature microglia (IM module, 4681 transcripts, enriched in E17 and P7 Saline), MIA immature microglia (MIA-IM module, 2816 transcripts, enriched in E17 and P7 MIA), Juvenile microglia (JM module, 2318 transcripts, enriched in P20 Saline and MIA), Adult microglia (AM module, 3276 transcripts, enriched in P60 Saline + CTRL, P60 MIA + CTRL and P60 MIA + MG-REP), and repopulated adult microglia (REP-AM module, 1,134 transcripts, enriched in P60 Saline + MG-REP) (clustering and representative genes in Fig. 2a and Supplementary Table 1). Interestingly, P60 Saline + MG-REP showed enrichment in a distinct REP-AM module, suggesting reprogrammed gene expression profiles of repopulated microglia (Fig. 2a). Furthermore, principle component analysis (PCA) of microglial transcriptome showed alteration of gene expression profiles by MIA at young ages (E17 and P7) (Supplementary Fig. 2a), which is consistent with previous reports [10]. At P60, only the Saline + MG-REP population appeared distinct compared with the other P60 groups (Supplementary Fig. 2a). We and others have recently identified the specific gene sets representing homeostatic (M0) and disease-associated/neurodegenerative microglia (DAM/MGnD) from mouse models of neurodegenerative disorders [27–29]. Analysis of microglial gene signature revealed that Saline + MG-REP possessed robust MGnD phenotype as determined by downregulation of M0 homeostatic microglia genes including *Tmem119*, and upregulation of DAM/ MGnD genes including Cstb, whereas MIA + MG-REP showed a homeostatic phenotype (Fig. 2b and Supplementary Fig. 2b). These results demonstrate an alteration in the composition of microglia during repopulation under MIA influence. The newly replenished microglia in Saline+MG-REP offspring show DAM/MGnD phenotype, which is characterized with higher phagocytic function, chemotaxis and proliferation, and is reminiscent of immature microglial phenotype [30]. On the other hand, microglia in MIA+MG-REP offspring did not show similar immature phenotype in MIA+MG-REP group, suggesting accelerated maturation in MIA+MG-REP group in accordance with recent publication [10]. Recent studies reported the in situ expansion of microglia from residual microglial pools after PLX5622 treatment [16, 17] and the CSF1R inhibitor resistant microglial pool was MAC2 positive [31], suggesting the source of repopulating microglia is also residual CSF1R inhibitor-resistant microglia in our study. To further evaluate this microglial repopulation source, we performed fate-mapping analysis. Thymidine analog 5-bromo-2′-deoxyuridine (BrdU) was repetitively injected from P1 to P20 in CTRL and MG-REP female offspring before PLX treatment, followed by repetitive injection of 5-Ethynyl-2′-deoxyuridine (EdU) from P43 to P59 to determine proliferating activity of microglia prior to the depletion (Fig. 2c). The cortical tissue sections were stained with IBA1, together with proliferation markers BrdU and EdU to assess if newly repopulated IBA1^+^EdU^+^ cells arise from IBA1^+^BrdU^+^ cells, which represent previously mitotic cells, or IBA1^+^BrdU^−^ cells, which represent previously non-proliferative cells (Fig. 2d and Supplementary Fig. 2C). The labeling efficiency of BrdU + IBA1 + was 40.54% and 35.13% of total IBA1 + cells in Saline + CTRL and MIA + CTRL group, respectively. The ratio of each of the populations (BrdU^+^EdU^−^, BrdU^+^EdU^+^, and BrdU^−^EdU^+^) was normalized to the total number of thymidine-analogs labeled IBA1^+^ cells (Fig. 2e). MIA reduced self-renewal reflected by lowered EdU^+^ cell number (Fig. 2e). Interestingly, previously non-proliferative BrdU^−^ cells (blue) were the main source of repopulation in MIA + MG-REP offspring (67%), whereas more microglia repopulated from previously proliferative BrdU^+^ cells (red and green, 60.8%) in Saline + REP offspring (Fig. 2e). Since Saline + MG-REP microglia show mitotic gene expression profile reminiscent of proliferating young and DAM/MGnD microglia (Fig. 2b and Supplementary Fig. 2b and Supplementary Table 1), this suggests that previously proliferative BrdU^+^ cells maintain mitotic activity after repopulation. On the other hand, MIA + MG-REP microglia are mainly from previously non-mitotic BrdU^−^ microglia, which may quickly revert to a non-proliferative gene expression phenotype similar to M0 homeostatic microglia (Fig. 2b and Supplementary Fig. 2b).

In order to further understand the MIA microglia gene expression modules in the context of neurodevelopmental disorders, we explored the common gene signatures in MIA microglia and gene modules identified in neurodevelopmental disorders. We compared the overlap of microglial modules to known schizophrenia, ASD, and intellectual disability risk genes, two de novo mutated gene sets in ASD patients, synaptic molecules, interacting partners of fragile X mental retardation protein (FMRP) and microglia modules from RNA-seq studies of ASD brains (Fig. 2f and Supplementary Table 2) using hypergeometric distribution analysis. The AM module, which was most enriched for MIA + CTRL microglia, showed enrichment for synaptic molecules, FMRP-, schizophrenia-, and ASD-implicated genes, while the MIA-IM cluster showed enrichment for genes with de novo mutations identified in human ASD and “M2” ASD prenatal genes (Fig. 2f). The REP-AM module overlapped with M1/M2 microglial gene lists, but lacked enrichment of ASD-associated genes. Importantly, there was no overlap between any modules and the 351 genes implicated in intellectual disability without ASD (Fig. 2f), suggesting that disruption of microglial transcriptome may not impact general intellectual ability. We next explored the overlap between AM and human ASD gene modules to determine which common genes underlie microglia associated molecular networks. We determined the GO terms represented by overlapping AM/M13, AM/M16, and AM/ M17 gene lists (Supplementary Table 3). Genes from the two most significant GO terms were input to ASD@Princeton (Fig. 2g), which identified microglial hubs including *Camk2* in AM/M13 and AM/M17 networks, a gene recently implicated in MIA [32] and also confirmed in FACS-isolated microglia [26]. Taken together, these newly identified microglia specific modules show a distinct microglial phenotype and repopulation source affected by MIA or PLX treatment.

### Enrichment of cellular protrusion and neuritogenic genes in MIA microglia

**Fig. 3:**
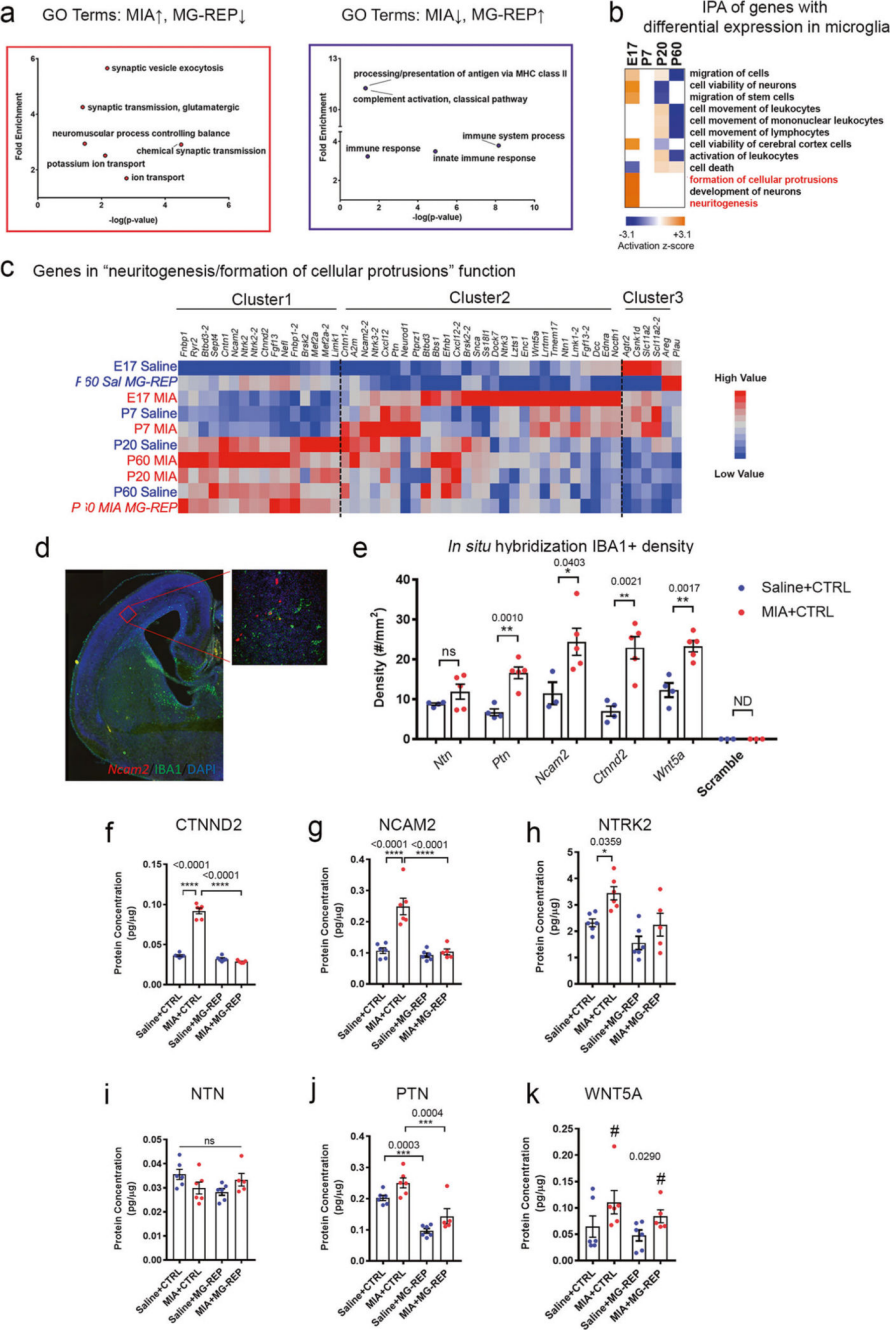
Microglia repopulation attenuates exacerbated production of cellular protrusion/neuritogenic factors from MIA microglia. **a** Gene ontology (GO) biological processes pathway analysis shows that MIA microglia increase synaptogenic functions while repopulated microglia recover homeostatic functions. Left (red): Top significantly enriched GO biological process terms increased by MIA and decreased by repopulation. Right (purple): Top significantly enriched GO biological process terms decreased by MIA and increased by repopulation. These GO findings were verified using GORILLA. **b** IPA of genes with differential expression in microglia between MIA versus Saline (RNA-seq data). Pathway analysis reveals MIA-induced upregulation of neuritogenic gene expression, specifically in developmental stages, based on activation *z*-score. Red denotes pathway activated in E17 MIA microglia. **c** Genes in “neuritogenesis/formation of cellular protrusions” function. Hierarchal clustering of gene sets based on relative expression values; red: high relative expression, blue: low relative expression. Cluster 1 represents genes increased in adult MIA microglia but reduced in MIA + MG-REP including *Ctnnd2*, *Ncam2*, and *Ntrk2*. Cluster 2 represents genes increased in immature MIA microglia including *Ncam2*, *Ntn*, *Ptn* and *Wnt5a*. Cluster3 represents genes decreased in immature MIA microglia including Plau. In situ hybridization (ISH) and immunofluorescence of E17 Saline or MIA offspring in the cortical plate region. **d** mRNA of cellular protrusion/ neuritogenic genes (*Ctnnd2*, *Ncam2*, *Ntn*, *Ptn,* and *Wnt5a*) were detected by florescent-labeled antisense cRNA probes (red) but not by scramble cRNA probe (not detected: N.D.), and the sections were immunostained for IBA1 (green) and DAPI (blue). **e** The number of IBA1 + cells expressing the cellular protrusion/neuritogenic genes were quantified in the cortical plate region. n = (4–5/2) male mice/ litters per molecule for Saline and MIA, *n* = 3 for scramble control probe. *p < 0.05, **p < 0.01, ns denotes no significance, by unpaired Student *t* test. Graphs indicate mean ± s.e.m. ELISA verification of selected RNA-seq molecules: CTNND2 (**f**), NCAM2 (**g**), NTRK2 (**h**), NTN (**i**), PTN (**j**) and WNT5A (**k**) in acutely isolated microglia. MIA increases protein expression of cellular protrusion/neuriotgenic molecules in microglia that were normalized via repopulation. *n* = (6/4, 6/3, 5/3, 6/3) female mice/litters for P60 Saline + CTRL, MIA + CTRL, Saline + MG-REP and MIA + MG-REP. CTNND2: Prenatal treatment effect, *F*(1,19) = 157.1, *p* < 0.0001, Drug effect, *F*(1,19) = 262.7, *p* < 0.0001, Interaction effect, *F*(1,19) = 201, *p* < 0.0001, NCAM2: Prenatal treatment effect, *F*(1,19) = 23.76, *p* = 0.0001, Drug effect, *F*(1,19) = 26.29, *p* < 0.0001, Interaction effect, *F*(1,19) = 17.63, *p* = 0.0005, NTRK2: Prenatal treatment effect, *F*(1,18) = 13.99, *p* = 0.0015, Drug effect, *F*(1,18) = 12.45, *p* = 0.0024, Interaction effect, *F*(1,18) = 0.06203, *p* = 0.8061, NTN: Prenatal treatment effect, *F*(1,19) = 0.01669, *p* = 0.8986, Drug effect, *F*(1,19) = 0.8, *p* = 0.3823, Interaction effect, *F*(1,19) = 6.121, *p* = 0.0230, PTN: Prenatal treatment effect, F(1,19) = 10.31, p = 0.0046, Drug effect, F(1,19) = 52.02, p < 0.0001, Interaction effect, *F*(1,19) = 0.002927 *p* = 0.9574, WNT5A: Prenatal treatment effect, *F*(1,19) = 5.581, *p* = 0.0290, Drug effect, *F*(1,19) = 1.550, *p* = 0.2282, Interaction effect, *F*(1,19) = 0.0834 *p* = 0.7799, *p < 0.05, **p < 0.01, ***p < 0.001 and ****p < 0.0001 as determined by 2-way ANOVA (alpha = 0.05) with Tukey’s post-hoc. ^#^*p* < 0.05 for main effect of MIA. Graphs indicate mean ± s.e.m.

To understand the molecular change induced by microglial repopulation in MIA male offspring, we performed GO biological process analysis of MIA-altered, repopulationcorrected genes from the AM module. Interestingly, the most highly enriched GO terms of MIA-upregulated genes corrected in MIA + MG-REP were associated with synaptic vesicles and neurotransmission (Fig. 3a, left red box), while the most enriched GO terms of genes downregulated in MIA and corrected in MIA + MG-REP were associated with antigen presentation, complement activation, and innate immune response (Fig. 3a, right purple box). This leads us to the notion that MIA microglia have abnormally increased synaptogenic properties, while repopulated microglia regain homeostatic immune functions. We crossexamined the 46 genes in the top GO term group, “chemical synaptic transmission” (Fig. 3a, left red box), with RNA-seq data of wild type and *Cx3cr1*^GFP/+^ microglia purified by FACS (GSE53789) [26]. We confirmed that 45 out of the 46 genes were detected in FACS-purified microglial gene sets. These data show strong evidence that microglia unexpectedly express synaptogenic molecules and that increased gene expression related to synaptic process in MIA microglia is unlikely due to the contamination of neuronal cells, but could reflect aberrant MIA microglial function.

We next performed cross-sectional analysis of 14,225 microglial genes in MIA versus Saline microglia using Gene-wise Negative Binomial Generalized Linear Modeling with Quasi-likelihood F-testing (glmQLFit) [33], which revealed discrete and overlapping genes significantly altered by MIA at each age [Supplementary Fig. 3a and Supplementary Table 4: 666 (E17), 225 (P7), 312 (P20), 161 (P60) unique genes with *p* < 0.05 MIA versus Saline], showing lifelong disruptions of microglial transcriptome by MIA. GeneMANIA analysis [34] revealed unique functional signature of the overlapped genes between the different time frames (Supplementary Table 4, Sheet 2). To determine how microglial transcriptional changes contribute to their cellular function in MIA offspring, we performed Ingenuity Pathway Analysis (IPA) of biological processes using all significantly affected genes by MIA at each age (Fig. 3b, Supplementary Fig. 3a and Supplementary Table 4), which identified “formation of cellular protrusions”, and “neuritogenesis” in the brain as the most enriched pathways in E17 MIA microglia (Fig. 3b). Indeed, current knowledge of microglia physiology supports this notion. Acute activation of microglia after injury or upon pathogen exposure results in the secretion of inflammatory factors and the phagocytosis of damaged neurons [35]. Surprisingly, however, microglia could produce synaptogenic factors such as insulin-like growth factor-1, thrombospondine, or brainderived neurotrophic factor, leading to regeneration of neurons after the acute phase [36–38]. In order to better characterize these pathways, we determined the hierarchical clustering of the genes and identified three clusters (Fig. 3c, Supplementary Table 5). Cluster 1 contained genes increased in adult MIA microglia but reduced in MG-REP, including cell adhesion molecules *Ctnnd2* (catenin delta 2, a synaptogenic factor) [39], *Ncam2* (neuronal cell adhesion molecule 2, an enhancer of filopodia formation) [40], and *Ntrk2* (neurotrophic receptor tyrosine kinase 2). Cluster 2 contained genes increased in immature MIA microglia, including *Ncam2*, *Ntn1* (netrin1, an axon guidance molecule), *Ptn* (pleiotrophin, a secretory growth factor), and *Wnt5a* (wingless-type MMTV integration site family, member 5A, an axon guidance and synaptogenic molecule) [41]. Cluster 3 contained genes decreased in immature MIA microglia including Plau. To assess the validity of microglial expression of cellular protrusion factors, we performed in situ hybridization with male offspring at E17 combined with immunofluorescence for IBA1 (Fig. 3d). We were able to detect all of the tested cellular protrusion molecules in IBA1^+^ cells in the cortical plate region of E17 embryonic brains while negative control using the scramble RNA probe did not have signal (Supplementary Fig. 3b). The number of IBA1^+^ cells expressing *Ctnnd2*, *Ncam2*, *Ptn*, and *Wnt5a* in the cortical plate region of E17 embryonic brains was higher in the MIA offspring compared with Saline controls, validating the RNA expression change found in our RNA-seq data (Fig. 3e). We further validated the data by qPCR with FACS purified E17 microglia from mixed male and female offspring, and confirmed significant increase in mRNA level in *Ncam2* and *Wnt5a* in MIA microglia (Supplementary Fig. 3c). Finally, we examined the protein expression level of those molecules in microglia isolated from Saline and MIA ± MG-REP female offspring at P60 by ELISA. Consistent with the results from RNA-seq or in situ hybridization, we confirmed upregulation of CTNND2, NCAM2, and NTRK2 by MIA and their normalization by microglial repopulation at P60 (Fig. 3f–h). Although MIA did not increase protein expression level in NTN or PTN, there was significant reduction by PLX in PTN in both Saline + REP and MIA + REP offspring (Fig. 3i, j). MIA had effect on WNT5A protein level, however PLX did not have treatment effect (Fig. 3k). Reactive synaptogenesis associated with microglial activation after inflammatory events has been reported in various conditions such as experimental models of epilepsy [42–44], facial nerve axotomy [45], and ischemia [36]. To understand how such transcriptome and translational changes in MIA offspring alter the interaction of microglia at pre- and post-synapse formations, we analyzed the effect of MIA on synaptic density and microglial interactions with specific pre- and post-synaptic neuronal structures in the layer V of mPFC using immunohistochemistry (Supplementary Fig. 3d). We observed a significant increase of P2RY12^+^/IBA1^+^ microglial contacts with VGLUT2^+^ presynaptic axon terminals, and the subset of excitatory synapses labeled with VGLUT2^+^/PSD95^+^ in MIA + CTRL compared with Saline + CTRL male offspring at P60 (Supplementary Fig. 3e). This indicates a potentially enhanced microglial interaction with neuronal synaptic structures in layer V neuropil of mPFC in MIA. Total excitatory synaptic density, measured as the number of PSD95^+^ puncta [46], did not differ between Saline + CTRL and MIA + CTRL (Supplementary Fig. 3f). We found a trend of increased number of VGLUT2^+^ puncta in MIA + CTRL group and a significantly increased number of VGLUT2^+^/PSD95^+^ synapses in MIA + CTRL compared with Saline + CTRL (Supplementary Fig. 3f). Taken together, these results suggest that immune perturbation during pregnancy may drive microglial transcriptome and proteome toward increased interaction and synaptogenic support, whereas PLX treatment on MIA adult offspring reverse microglial aberrant function and recover their homeostatic phenotype.

### Microglia repopulation ameliorates MIA-induced changes in neurophysiological properties

**Fig. 4:**
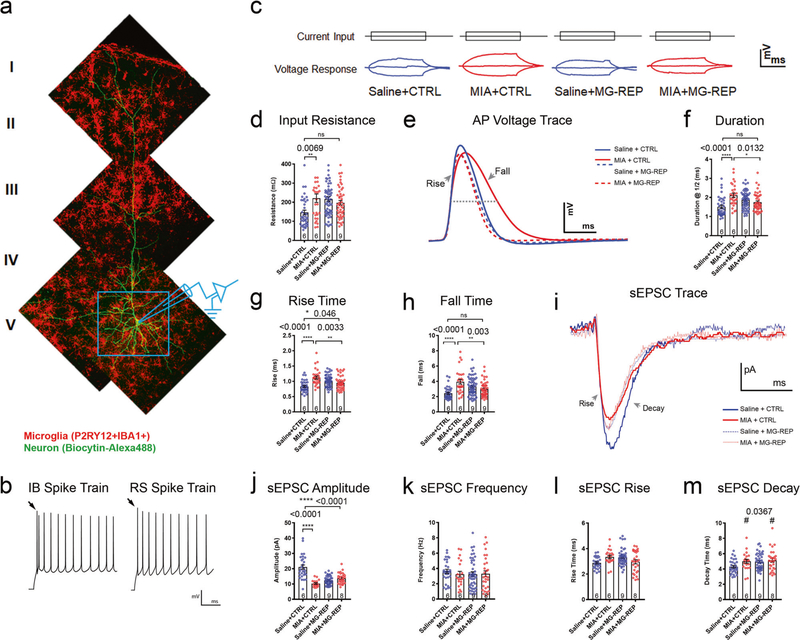
MIA-induced alterations to intrinsic membrane and excitatory synaptic current properties are ameliorated by microglial repopulation. **a** Experimental design: Whole-cell patch-clamp recordings were performed on layer V pyramidal neurons. Merged confocal *z*-stacks showing biocytin-filled neuron (green) and P2RY12^+^IBA1^+^ microglia (red) from layer V to layer I of the medial prefrontal cortex (mPFC) of male mouse. **b** Representative repetitive action potential firing responses to a +120 pA current step of an intrinsically bursting (IB) and a regular spiking (RS) layer V pyramidal neuron. Note the initial action potential doublet in the IB cell (arrow, left), compared with the initial single action potential in the RS cell (arrow, right). Scale bar: *y*-axis = 20 mV, *x*-axis = 250ms. **c** Voltage responses (bottom traces) to 200ms depolarizing (+20 pA) and hyperpolarizing (−20 pA) current steps (top traces) in representative neurons from each of the four groups. Scale bar: *y*-axis = 4 mV, *x*-axis = 100 ms. **d–h**: Intrinsic properties of layer V IB cells: **d** Mean input resistance (Rn) Prenatal treatment effect, *F*(1,164)= 3.4573, *p* = 0.0648, Drug effect, *F*(1,164) = 2.6039, *p* = 0.1085, Interaction effect, *F*(1,164)= 10.40917, *p* = 0.0015. **e** Representative traces of action potentials from layer V pyramidal neurons in slices prepared from Saline + CTRL (blue), MIA + CTRL (red), Saline+ MG-REP (blue dotted) and MIA + MG-REP (red dotted) mice, which revealed MIA offspring displaying significantly slower AP kinetics (**f–h**) that were recovered via repopulation. Scale bar: *y*-axis = 20 mV, *x*-axis = 1 ms. **f** Mean duration at half maximal amplitude of the action potential (denoted by horizontal dotted line in **e**). Prenatal treatment effect, *F*(1,168) = 9.570, *p* = 0.0023 Drug effect, *F*(1,168) = 0.001748, *p* = 0.9667, Interaction effect, *F*(1,168) = 21.06908, *p* < 0.0001. **g** Mean rise, Prenatal treatment effect, *F*(1,166) = 12.44126, *p* = 0.0005, Drug effect, *F*(1,166) = 0.02641, *p* = 0.8711, Interaction effect, F(1,166)= 29.5484 *p* < 0.0001. **h** Fall time of the action potential. Prenatal treatment effect, *F*(1,166) = 10.2777, *p* = 0.0016 Drug effect, *F*(1,166)= 0.076, *p* = 0.7831, Interaction effect, *F*(1,166)= 25.099, *p* < 0.0001. Neurons in each group: *n* = (38/6/4, 30/6/4, 60/9/6, 45/9/7) cells/male mice/litters for (Saline + CTRL, MIA + CTRL, Saline + MG-REP, MIA + MG-REP). **i** Representative waveforms of averaged spontaneous excitatory postsynaptic current (sEPSC) in Saline+ CTRL (blue), MIA + CTRL (red), Saline + MG-REP (blue dotted), and MIA + MG-REP (red dotted) layer V neurons indicating significant MIA effect in certain sEPSC properties. Scale bar: *y*-axis= 4pA, *x*-axis = 10ms. sEPSC of layer V IB cells: **j** Mean sEPSC amplitude, Prenatal treatment effect, *F*(1,114)= 27.6523, *p* < 0.0001, Drug effect, *F*(1,114) = 9.811, *p* = 0.0022, Interaction effect, *F*(1,114) = 47.32085, *p* < 0.0001. **k** Mean sEPSC frequency, Prenatal treatment effect, *F*(1,121)= 0.20, *p* = 0.6555 Drug effect, *F*(1,121)= 0.216094, *p* = 0.6463, Interaction effect, *F*(1,121) = 0.4017, *p* = 0.5274. **l** Mean sEPSC rise time, Prenatal treatment effect, *F*(1,121) = 0.2817, *p* = 0.5966 Drug effect, *F*(1,121)= 0.3228, *p* = 0.8577, Interaction effect, *F*(1,121)= 11.2053, *p* = 0.0011. **m** Mean sEPSC decay time, Prenatal treatment effect^#^, *F*(1,119) = 4.4625, *p* = 0.0367 Drug effect, *F*(1,119) = 2.9849, *p* = 0.0866, Interaction effect, *F*(1,119) = 1.4879, *p* = 0.2250. Neurons in each group: *n* = (29/6/4, 20/6/4, 46/9/6, 30/8/6) cells/male mice/litters in the same order as **d–h**. *p < 0.05, **p < 0.01, ***p < 0.001 and ****p < 0.0001, ns denotes no significance; determined by 2-way ANOVA (alpha= 0.05) and Tukey’s post-hoc. Graphs indicate mean ± s.e.m.

Microglia migrate along callosal and subcerebral tracts to cortical deep layers at the early postnatal phase, and support survival of layer V pyramidal cells during development [47]. The deep layer of the mPFC has also been implicated in aberrant neuronal circuitry and synchrony in MIA [21]. To determine if microglial transcriptomic and proteomic changes during MIA have an effect on neuronal activity in this region, we performed whole-cell patch clamp recordings from layer V pyramidal neurons in slices of the mPFC from P60 male mice (Fig. 4a). Neurons were classified as intrinsically bursting (IB) or regular spiking (RS) as described in “Methods” (Fig. 4b). Layer V contains excitatory neurons and synapses involved in both corticocortical and cortico-subcortical circuitry [48]. IB neurons comprise a relatively small subset (10–20%) of cortical neuronal projections to subcortical structures such as the striatum and thalamus, whereas RS neurons give rise to projections to ipsilateral or contralateral cortex or striatum [48, 49]. There were no significant effects of MIA on the electrophysiological properties of RS neurons (Supplementary Figs. 4a–l); thus, all the rest of data presented are from IB neurons. IB neurons in MIA + CTRL offspring showed significantly increased input resistance (Rn), relative to Saline + CTRL offspring; this significant difference was not seen in MIA + MG-REP compared with Saline + CTRL neurons (Fig. 4c, d). Neurons from MIA + CTRL mice exhibited significantly prolonged action potential (AP) kinetics (Fig. 4e), including increased duration at half amplitude (Fig. 4f), increased AP rise time (Fig. 4g), and increased AP fall time compared with Saline + CTRL (Fig. 4h). These kinetic changes were reversed in MIA + MG-REP neurons, which did not differ from Saline + CTRL neurons except AP rise time (Fig. 4f–h). There were no effects of MIA on other intrinsic firing properties. In addition, PLX treatment show increase in input resistance, duration, AP rise time and fall time in Saline + MG-REP neurons compared with Saline + CTRL neurons. We next measured spontaneous excitatory postsynaptic currents (sEPSCs) and tetrodotoxin-insensitive miniature excitatory postsynaptic currents (mEPSCs). MIA resulted in a significant decrease in the mean sEPSC amplitude in MIA + CTRL compared with Saline + CTRL neurons, which was not reversed by MG-REP (Fig. 4i, j). MIA had no effect on sEPSC frequency or rise time but had an effect on decay time (Fig. 4k–m). Furthermore, sEPSCs amplitude in Saline + MG-REP neurons showed significant reduction compared with Saline + CTRL neurons, suggesting possible functional change in replenished microglia. This could be due to the reduction in homeostatic gene expression in Saline + MG-REP microglia, particularly Cx3cr1 (Fig. 2b). Indeed, recent study reports that deletion of Cx3cr1 reduces EPSC amplitude in CA1 synapses [50]. Induction of DAM/MGnD phenotype in Saline + MG-REP but not in MIA + MG-REP microglia could affect development of glutamatergic synapses. There were no effects of MIA or PLX on spontaneous inhibitory postsynaptic currents (sIPSC) of IB cells (Supplementary Figs. 4m–p). Interestingly, there was no change in amplitude or kinetics of mEPSCs, but there was a significant increase in the mEPSC frequency in the MIA group; an effect that was not reversed by MG-REP (Supplementary Figs. 4q–t). In summary, IB but not RS layer V pyramidal neurons in MIA offspring exhibit functionally significant changes in intrinsic and synaptic response properties, including slower AP kinetics and reduced amplitude sEPSCs, effects that were partially reversed by microglial repopulation.

### Microglia repopulation reverses increased basal dendritic spine density and enhanced microglia–neuron interactions in MIA offspring

**Fig. 5:**
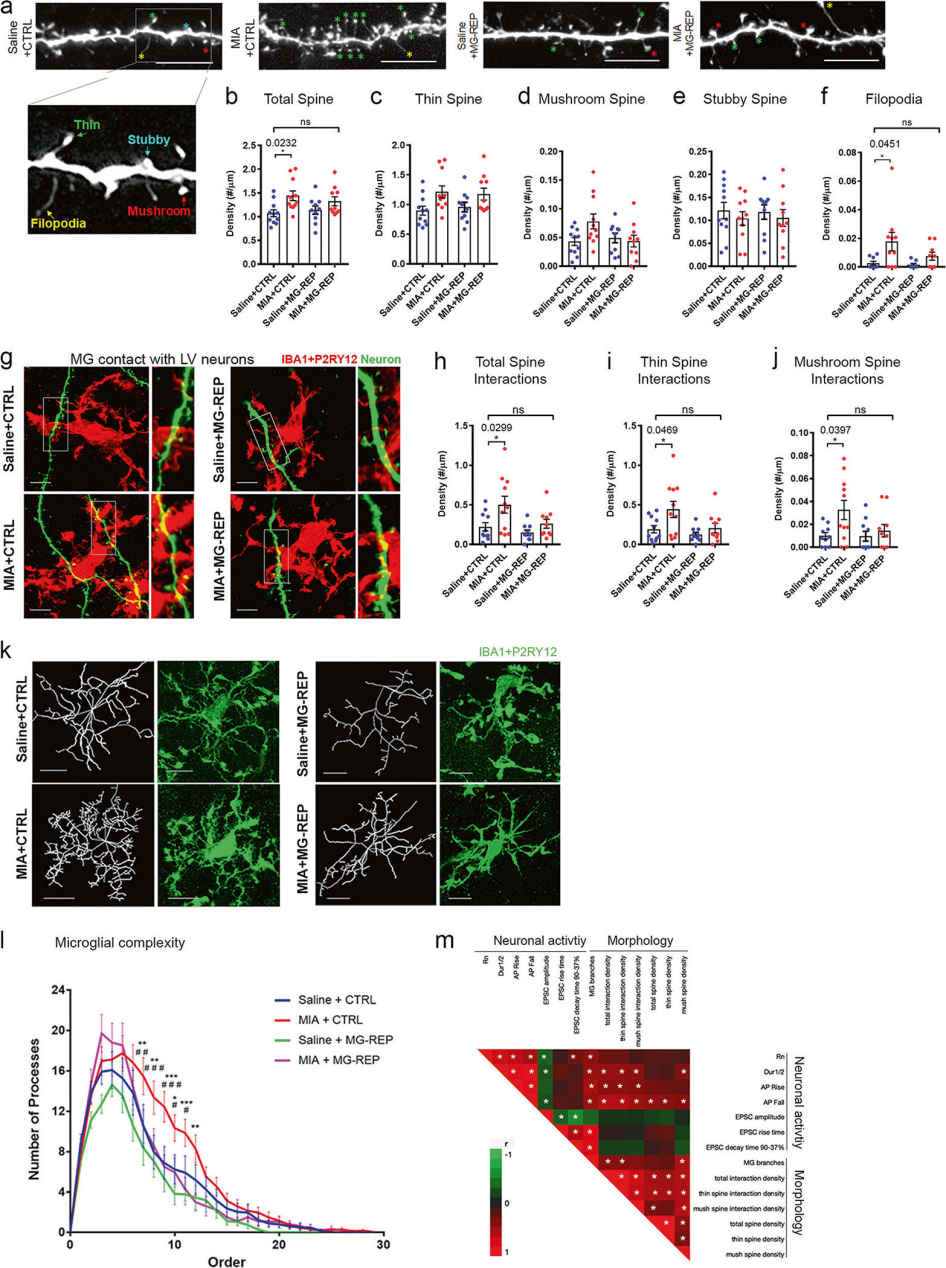
CSF1R inhibitor treatment reverses increased basal dendritic spine density and enhanced microglia-neuron interactions in MIA offspring. **a** Representative confocal images of biocytin-filled basal dendrites and spines of layer V pyramidal neurons of male mice. Scale bar = 7 μm. Asterisks mark each spine subtype: yellow =filopodia, red = mushroom, blue = stubby, green = thin. **b-f** MIA induces an increase in spine densities of layer V IB cells: MIA offspring display an increase in total, Prenatal treatment effect, *F*(1,38) = 9.7002, *p* = 0.035 Drug effect, *F*(1,38) = 0.08105, *p* = 0.7775, Interaction effect, *F*(1,38) = 1.67, *p* = 0.3082 (**b**) and filopodia Prenatal treatment effect, *F*(1,32) = 7.0552, *p* = 0.0122 Drug effect, *F*(1,32) = 2.07424, *p* = 0.1595, Interaction effect, *F*(1,32) = 1.276, *p* = 0.267 (**e**) spine densities, which is absent between Saline and MIA MG-REP groups. Thin (**c**), mushroom Prenatal treatment effect, *F*(1,38) = 2.3087, *p* = 0.1435, Drug effect, *F*(1,38) = 1.9774, *p* = 0.1678, Interaction effect, *F*(1,38) = 4.1241, *p* = 0.0493 (**d**) or stubby, Prenatal treatment effect, *F*(1,38) = 0.8267, *p* = 0.369 Drug effect, *F*(1,38) = 0.004168, *p* = 0.9489, Interaction effect, *F*(1,38) = 0.01588, *p* = 0.9007 (**f**) spine densities are unchanged. **g** Representative images of microglia (red) interacting with basal dendritic spines (green) of layer V pyramidal IB cells. Interaction was analyzed as described in Supplementary Figs. 5b–f. Scale bar = 4 μm. MIA offspring display an increase in microglia-spine interactions, which is absent between Saline and MIA MG-REP groups: Number of microglial interactions with total, Prenatal treatment effect, *F*(1,38) = 7.9465, *p* = 0.0076, Drug effect, *F*(1,38) = 5.09753, *p* = 0.0298, Interaction effect, *F*(1,38) = 1.4105, *p* = 0.2423 (**h**), thin Prenatal treatment effect, *F*(1,38) = 6.2423, *p* = 0.0169, Drug effect, *F*(1,38) = 5.089, *p* = 0.0299, Interaction effect, *F*(1,38) = 1.5544, *p* = 0.2201 (**i**) and mushroom spines Prenatal treatment effect, *F*(1,37) = 5.5939, *p* = 0.0234, Drug effect, *F*(1,37) = 2.5284, *p* = 0.1203, Interaction effect, *F*(1,37) = 2.3474, *p* = 0.1340 (**j**). **b–f, h–j**, Dendrites at least 95 μm distant from cell soma were evaluated. *n* = (11/8/5/3, 11/8/5/4, 10/8/5/3, 10/4/3/3), dendrites/cells/male mice/litters for (Saline + CTRL, MIA + CTRL, Saline + MG-REP, MIA + MG-REP). *p < 0.05, **p < 0.01, ***p < 0.001, and ****p < 0.0001, ns denotes no significance; determined by 2-way ANOVA (alpha = 0.05) and Tukey’s post-hoc. Graphs indicate mean ± s.e.m. **k** Representative P2RY12^+^IBA1^+^ microglia ×40 original magnification. Scale bar = 10 μm. **l** Microglial branch tracings shows that microglia repopulation normalizes MIA-induced hyperramification. Colors of trace points denote branch order. *n* = (25/6/4, 36/5/4, 24/6/4, 20/6/5) (cells/male mice/litters) for Saline + CTRL, MIA + CTRL, Saline + MG-REP, MIA + MG-REP. One, two, and three * or # symbols denote *p* < 0.05, 0.01, 0.001, between MIA + CTRL vs. Saline + CTRL or MIA + MG-REP by 2-way ANOVA and Tukey’s post-hoc, respectively. **m** Spearman’s correlation analysis of neuronal activities and morphological data from within-cell comparisons. Color map of rho values for positive and negative correlations from red (1) to green (−1). The properties of neuronal activities from IB cells include Rn, Dur½, AP rise, AP fall, sEPSC amplitude, sEPSC rise time, and sEPSC decay time. The properties of morphological assessments include MG branches (at 9th order), total spine interaction (with microglia) density (per dendritic length), thins spine interaction density, mush(room) spine interaction density, total spine density, thin spine density and mush(room) spine density. * denotes *p* < 0.05 as determined by Pearson’s correlation.

Neuronal plasticity and connectivity depend on changes in dendritic spine morphology and density. Our results showing aberrant production of cellular protrusion/ neuritogenic factors from MIA microglia suggests possible spine dysgenesis in MIA offspring. Therefore, we assessed the effects of MIA and microglial repopulation on dendritic spine density and their sub-classifications of basal dendrites in biocytin-filled and electrophysiologically characterized layer V IB cells in male offspring at P60 as previously described [51] (Fig. 5a). Microglia repopulation did not alter the spine characteristics in Saline offspring (Fig. 5b–f). Notably, total and filopodia spine densities were significantly increased in MIA + CTRL offspring but not for stubby or mushroom spine densities (Fig. 5b–f and Supplementary Fig. 5a). Microglia repopulation (MIA + MG-REP) normalized the difference seen in total and filopodia spine density between Saline + CTRL and MIA + CTRL offspring (Fig. 5b, f). Spine dysgenesis is a common abnormality observed in neurodevelopmental disorders, including increased spine density reported in MIA offspring [52, 53]. On the other hand, Coiro et al. has previously reported the reduction of spine densities in layer V pyramidal neurons in the somatosensory cortex at P17–19 and 3 months of age, which may be due to the different regimen they used for the MIA induction injecting poly(I:C) at E12.5 in comparison to E9.5 in our study and the difference in the tested brain region [54]. Importantly, microglia have an established role in synaptic plasticity [55], and their contacts to dendritic shafts induce filopodia formation [56, 57]. Thus, morphological changes observed in dendritic spines may be due to their interaction with microglia. We characterized microglial interactions with biocytin-filled basal dendritic spines of layer V IB pyramidal neurons of male offspring (Fig. 5g and Supplementary Figs. 5b–e). MIA increased microglial interactions with total, thin and mushroom spines in MIA + CTRL compared with Saline + CTRL group, while microglial repopulation (MIA + MG-REP) negated this effect (Fig. 5h–j). Microglial interactions with dendritic spines were further divided into three subtypes based on the proximity of microglial processes to dendritic spines (Supplementary Fig. 5f, g). All types of microglia–spine interactions were increased in MIA + CTRL compared with the Saline + CTRL group, but there was no differences between Saline + CTRL and MIA + MG-REP groups (Supplementary Figs. 5h–j). To validate the effect of MIA microglia on spine formations, we performed confocal microscopic live-imaging of primary mouse cortical neurons co-cultured with FACS-purified microglia isolated from E17 Saline + CTRL or MIA + CTRL offspring, the time point we saw robust changes in microglial gene expression by MIA, at days in vitro 19 (DIV19). Interestingly, co-cultured MIA microglia significantly increased the proportion of filopodia spines as compared with co-cultured saline microglia (Supplementary Figs. 5k, l), reflecting the in vivo finding of enhanced filopodia formation in MIA offspring. These findings imply morphological changes in layer V pyramidal neurons, which are paralleled with their interaction with microglia in MIA offspring and reversed after PLX treatment.

Microglia, as immune surveyors, display heterogeneous morphologies that reflect their response to the environmental cues [5]. Changes we observed in gene expression profiles of microglia, such as enhanced formation of cellular protrusions, and closer interactions between microglia and dendritic spines may correspond with morphological changes in MIA microglia. To determine MIA effect on microglial morphology and whether MIA phenotype is retained in repopulated microglia, we analyzed the morphology of microglia from P60 CTRL and MG-REP male offspring in layer V of the mPFC. Confocal imaging and three-dimensional analysis of microglia with P2RY12 and IBA1double-immunofluorescence revealed complex branches up to the 25th order (Fig. 5k). We found significantly increased ramification in MIA+CTRL compared with Saline+CTRL microglia (Fig. 5k top and bottom left panels) via three-dimensional tracing and quantification of microglial branches [58], suggesting that MIA induces heightened complexity in microglial branching (Fig. 5l, red versus blue, increased branch number at 8th–12th orders). Interestingly, repopulated microglia recovered normal branching morphology in MIA + MG-REP (Fig. 5k, bottom right panels and 5l, red versus magenta).

Finally, to determine how morphological changes in microglia and dendritic spines and their interactions may affect synaptic property, we performed pairwise Spearman’s correlation analyses of morphological (microglia and neuron) and neurophysiological properties using the same biocytin-filled IB cells and their corresponding microglial interactions (Fig. 5m, Supplementary Table 6). The number of microglial branches was positively correlated with microglial spine interactions (total and thin spines) and mushroom spine density. Furthermore, increased microglial branches were positively correlated with changes in intrinsic membrane properties (Rn) and kinetics of action potentials (duration, rise, fall), and kinetics of excitatory postsynaptic activity (EPSC rise and decay), reflective of postsynaptic neuronal response (Fig. 5m). In addition, microglial interaction with dendritic spines (total, thin and mushroom spines) was positively correlated with kinetics of action potentials (duration, rise, fall).

Collectively, these findings suggest that MIA induces aberrant microglial morphological structure, microglia-spine interaction, and synaptogenesis, which were partially corrected by microglial repopulation. Spearman correlation analysis reveals synergistic positive correlation of microglial branching with their synaptic interactions, intrinsic firing kinetics, and EPSC properties of interacting IB cells.

## Discussion

Our results reveal that microglia play a pivotal role in altering neuronal development and the abnormal behaviors induced by MIA. Establishing the link between prenatal neuroimmune abnormalities and neuronal developmental defects has been challenging. Here, we systematically characterized the role of microglia in the MIA phenotype under microglial repopulation using behavioral, electrophysiological, cellular, and molecular approaches. We demonstrated that MIA leads to lifelong disruptions of microglial transcriptome and microglia–synapse interactions, which coalesce with synaptic defects including increased immature spine density and enhanced repetitive behavior, and social deficits (Supplementary Fig. 5m). VGLUT2^+^/PSD95^+^ puncta in layer V cortex represents a subset of synapses, formed by axons originating mostly from neurons in the thalamus, which make up ~90% of VGLUT2^+^ projections to the mPFC [59]. Our data show that specific subsets of thalamo-cortical input (VGLUT2^+^ synapses) and putative cortico-thalamic output neurons (IB cells) in the mPFC are selectively affected in MIA.

Microglial repopulation may be a potential therapeutic approach for neurodevelopmental disorders associated with prenatal immune insults. PLX treatment targeting CSF1R reverses synaptic abnormality including increased immature spine densities and abnormal behavior in MIA offspring, suggesting that inflammation-induced microglial and synaptic dysfunction may be plastic and reversible. A recent single cell RNA-seq study of repopulated microglia at day 5 of the repopulation period also found that repopulated microglia differ from the original microglia [16]. This indicates that repopulation may facilitate the functional correction of MIA microglia. Indeed, we observed PLX treatment downregulates the microglial protein expression level of cellular protrusion/neuritogenic factors, and synaptogenic property and restored homeostatic immune function. Aberrant neuronal support by exacerbated secretion of those factors from MIA microglia could be detrimental during neural development, which requires precise programming and wiring of neural networks. Our data revealed elevated mRNA expression of cellular protrusion molecules by RNA-seq analysis, in situ hybridization, qPCR, and elevated protein levels in MIA microglia compared with Saline microglia. Augmented transcription and translation of specific synaptic genes may be a novel convergence point of MIA-induced and genetic neurodevelopmental diseases. ASD related gene mutations such as *FMR1*, *PTEN*, *EIF4E*, and *CYFIP*, increase gene transcription and protein translation, and are closely associated with increased number of immature synapses (filopodia-like protrusions) and synaptic strength [60, 61]. Among enriched cellular protrusion/neuritogenic molecules in our MIA microglial molecular profiling, *Ctnnd2*, *Enc1*, *Brsk2*, *Ntrk2*, *Ntrk3*, and *Ryr2* are also FMRP-targeted genes [62, 63]. Moreover, we identified significant gene expression enrichment between AM genes and subsets of molecules found in ASD and schizophrenia. This overlap suggests that a common mechanism may exist between the microglial dysfunction induced by MIA and neurodevelopmental disorders, culminating in altered neuronal circuit formation and abnormal behaviors.

In summary, our results indicate the plasticity of prenatally-immune-perturbed microglia and neurons and the potential therapeutic application of CSFR1 inhibitors to MIA-related neurodevelopmental disorders.

## Material and methods

### Animals

Male and female C57BL6/J mice (Jackson Laboratory) mice were kept on a 12-h light/12-h dark schedule with access to chow ad libitum. Pregnant mothers were singlehoused after timed mating. Offspring were weaned at P21 and group-housed with 2–5 same sex littermates per cage. For all behavior studies, only male mice were used and separated litters for MIA and saline group were used due to prenatal injection of pregnant mothers. The number, sex, and age of animals for each experiment were listed in Figure Legends. No statistical methods were used to predetermine sample size, and randomization of samples was not required. All animal procedures followed the guidelines of the National Institutes of Health Guide for the Care and Use of Laboratory Animals and were approved by the Boston University Institutional Animal Care and Use Committee.

### Neuron and microglia co-culture preparation

Murine primary cortical neurons were isolated as previously reported [64]. Briefly, cortex of the embryonic mice at E16.5 were isolated in Hibernate-E medium (ThermoFisher) at 4 °C. Tissues were cut into 1-mm^3^ pieces and incubated with 0.25% trypsin-EDTA solution (ThermoFisher) for 15 min at 37 °C and dissociated into single cells by gentle trituration. Neurons were then plated at a density of 1.0 × E4 cells per chamber on poly-D-lysine pre-coated 35 mm glassbottom dishes with four micro-chambers (Ibidi) with DMEM (ThermoFisher) containing 20% fetal bovine serum in 37 °C 5%CO_2_ with controlled humidity incubator. Four hours later, the medium was refreshed with neuronal culture medium (Neurobasal medium, 2% B27, 2 mM Glutamax). Media were changed by half every two days. At days in vitro (DIV) 3, neurons were infected with CMV-rtTA (Addgene) and TetO-hNGN2-EGFP (Addgene) lentivirus. One day after the infection, 2 μg/ml doxycycline (SigmaAldrich) was added in media to induce neuron maturation and GFP expression. At DIV14, embryonic murine microglia were isolated and cultured in Neurobasal medium (ThermoFisher) supplemented with 20 ng/ml CSF1 (R&D System) as previously described using FACS as CD11b ^+^FCRLS^+^LY6C^−^ cells except from E17.5 embryonic brains derived of Saline or poly(I:C) injected dam (n = 10 pooled embryos from 1–2 litters). Microglia were plated at a density of 2.5 × 10^4^ cells per chamber with DIV14 neurons. Neurobasal medium was refreshed two days later with 20 ng/ml murine CSF1 and 2 μg/ml doxycycline.

### Maternal immune activation and microglia depletion/repopulation

#### Maternal immune activation

Eight to twelve weeks old adult female C57BL6/J mice were subjected to timed-mating with male C57BL6/J mice. One to two female mice were placed in the male’s cage between 2 and 3 p.m., and female mice were checked for presence of a vaginal plug 16–17 h later. On embryonic day 9.5 (E9.5), female mice were intraperitoneally (ip) injected with 20 mg/kg poly(I:C) in potassium salts (Santa Cruz Biotech, supplied at ~10% of the total weight of the salt. Dosage was based on the weight of poly(I:C) itself, calculated for the exact percentage for each lot [65]. E9.5 corresponds to the middle of the first trimester in terms of human developmental biology, at which time studies have reported increased likelihood of prenatal infection being associated with neurodevelopmental alterations in humans [2]. This paradigm has been confirmed to lead to physiological markers of immune response in mothers and microglial alterations in offspring [66].

#### Microglia depletion and repopulation

Microglia depletion and subsequent repopulation was achieved through administration of the colony stimulating factor 1 receptor (CSF1R) inhibitors PLX5622 (1200 ppm delivering daily doses of 168 mg/kg) (Plexxikon, Inc., mixed into AIN-76A standard chow by Research Diets, Inc.) from postnatal day P21–42 (MG-REP group) [14, 15].

### Behavioral testing

All behavioral tests were performed in an empty testing room within the animal vivarium, during the light cycle between the hours of 9 a.m. and 6 p.m. For all tests, animals were habituated in the test room for at least 1 h before the start of testing. A test-free period of 5–7 days was used between behavioral tests. A white noise machine was used on low setting for all tests except the three-chamber social interaction test for which communication is important. The lighting, humidity, temperature, and ventilation were kept as constant as possible in the testing room. The experimenter was not present in the room during any of the tests. When possible, the experimenter performing behavior tests was blind to the treatment group; PLX-containing chow is differently colored than control chow, thus it was impossible to blind the experimenter to the difference between chow groups. All behavioral analyses were performed in a blinded manner. Animal number is reported in Figure legends.

#### Self-grooming test

The self-grooming test was performed as described [3]. Briefly, mice were placed individually in a 6.5-cm diameter × 12-cm tall clear glass beaker covered with a filter top. After a 10 min habituation period in the cage, mice were video-recorded for 10 min. Beakers were only used once per day and rinsed with warm water and soap and thoroughly dried at the end of each day. Videos were analyzed by an experimenter blinded to the treatment groups. Self-grooming was considered any time the mouse touched themselves with their mouth or paws.

#### Three-chamber social interaction test

The social interaction test was performed as described [67, 68]. An opaque plexiglass three-chambered apparatus was custom-designed by our lab and fabricated by Boston University Scientific Instrument Facility. A mouse was placed in the middle chamber with door-blocking walls in place, and left to habituate for 5 min. Then, an unfamiliar same age, sex, and weight-matched mouse (within 5 g) mouse was placed in a clear perforated plexiglass cylinder in one of the side chambers, to the right or left of the entranceway. An identical empty cylinder was placed in the opposite side chamber. The chamber wall dividers were removed, and the test mouse was allowed to explore freely for 10 min while being video recorded. The mouse was then placed in a new empty cage and the apparatus was cleaned thoroughly with 20% ethanol. The time spent in each chamber, number of entries into each chamber, was videotracked using Ethovision XT14 software.

#### Ultrasonic vocalizations test

USV testing was performed modified from Malkova et al. [3]. Males were isolated on P62 and housed singly for 7 days prior to USV testing, on P68. Mice that underwent USV testing did not undergo social approach testing. In order to establish sexual experience to facilitate USV, an adult female mouse was placed in each male’s cage for 15 min per day, for 4 days prior to USV testing (between P64–P67). On the day of testing, male mice were placed in a clean cage in a sound attenuating chamber for habituation for 10 min. An unfamiliar, age-matched, estrous female was then added to the cage and USVs were recorded for 3 min. Females were assessed for estrus cycle using visual inspection, as previously established [3]. Female mice were only used once per day. USVs were recorded using an Avisoft Biocaoustics Condenser ultrasound microphone (suspended 20 cm above the cage floor) and Avisoft-RECORDER. USVs were automatically analyzed using Avisoft-SASLab Pro, after a fast Fourier transformation (FFT-length: 512, frame: 100%—hamming window, time window overlap: 75%), using a threshold-based algorithm with a hold-time of 30 ms. The total number of vocalizations produced was recorded.

### Microglia isolation and whole-genome RNA expression analyses

#### Cell Isolation procedure + RNA extraction

Primary microglia were isolated from the offspring of saline or poly(I:C)-treated mothers under sterile conditions, using CD11B MicroBeads (Miltenyi Biotec) according to the manufacturer’s protocol. CD11B^+^ cells (1–4 million cells per sample, where “sample”= one pool of brains from one litter at E17, one pool of three brains from P7, or an individual brain for P20 and P60 mice) were immediately lysed in Qiazol (Qiagen) and frozen at −80 °C. RNA was extracted using the miRNeasy kit (Qiagen), and quantified using a Nanodrop (ThermoFisher, 260/280 = 1.9–2.1). A Qubit (ThermoFisher) was used to confirm concentration in a portion of samples. RNA concentration and purity were confirmed by Bioanalyzer 2100 (Agilent Technologies). All samples had a RIN between 8.5 and 10.

#### RNA sequencing

Truseq (Illumina) non-stranded cDNA libraries were constructed from poly(A)-enriched mRNA. RNA sequencing was performed using Illumina HiSeq2000 at the Massachusetts Institute of Technology (MIT) MicroBio Center using 40-base pair single end reads, with at least 20 million reads per sample (one sample = library constructed from RNA isolated from the microglia of one whole brain (P20 and P60 groups), pooled whole brains (P7 groups) or whole brains of one litter (E17 embryos). The Bioconductor Rsubread package in the R programming language was used to align reads to the mouse genome and obtain feature counts [69]. To build an index to map the reads, the R function “buildindex” was used with the mm10 Mus musculus reference genome. To align the reads, the Rsubread function “Align” was used with the “seed- and-vote” paradigm for read mapping, which reports the largest mappable region for each read. This generated (.bam) files for read summarization. For read summarization, an annotation file (.gtf) from UCSC genome browser (https://genome.ucsc.edu) was used, with the Rsubread function “featureCounts,” to assign reads to genomic features of interest once they are mapped to the reference genome. The BatchQC pipeline [70] was used to check the effect of multiple batches during sequencing and technical bias due to sequencing being processed in different batches; no such effects were found. We evaluated litter and gender as possible confounding effects. Based on this analysis and our balanced experimental design, we concluded that there was no evidence that these variables modified the effect of treatment or any of our downstream conclusions. Principal component analysis was run to compare variability between different groups per age per condition using BatchQC. Comparison analyses between groups were performed in EdgeR [33]. A generalized linear model with Quasi Likelihood F (QL-F) testing (glmQLFit) was used for paired and group comparisons. Feature count data was grouped, normalized, and dispersions were estimated. Data was then filtered to retain all genes with counts per million (CPM) of 2 or above in at least three replicates (out of 30 total replicates), yielding 14,225 genes. For paired comparisons, an exact test was performed and genes with *p* < 0.05 were considered significantly different. For group comparisons, a model matrix was designed according to condition [poly(I:C)] and treatment (MG-REP), data was normalized, dispersions were estimated, and glmQLFit was performed. For within-group age comparisons, the “makeContrast” R function was used. Genes with p < 0.05 from respective comparisons were considered significant. *ComplexHeatmaps* R package was used to perform K means clustering (*k* = 5) and create a heatmap of clustered genes. The optimal cluster number was determined based on the Elbow method. For IPA (Qiagen bioinformatics) of RNAseq data, gene lists of exact test results (MIA versus Saline) were uploaded to IPA (with corresponding *p* values and fold changes), and core analyses were run to determine upstream regulators, canonical pathways, biological functions, and diseases associated with each comparison. The identified upstream pathways or biological processes showed significant enrichment with *p* < 0.01 in at least one of the four or five comparison groups. Multiplot Studio (GenePattern Archive) was used to create volcano plots, Venny 2.1 (BioinfoGP, http://bioinfogp.cnb.csic.es/tools/venny/) was used to create Venn diagrams.

#### Assessment of microglia purity

To verify purity of RNA-seq across ages and conditions, we assessed expression of cell-type specific markers (derived from Barres dataset of mouse neurons, astrocytes, oligodendrocytes, endothelial cells, and microglia) [25], which demonstrate average of 98% purity.

The following genes: *Dcx*, *Npas4*, *Npy*, *Reln*, *Tubb3* (neurons), *Mag*, *Mbp*, *Mog*, *Ppapdc1a*, *Sox10* (oligodendrocytes), *Aqp4*, *Gfap*, *Gdpd2*, *Plcd4*, *Slc7a2*, (astrocytes), *Emcn*, *Ocln*, *Pecam1*, *Slc16a4*, *Tek* (endothelial cells), *C1qa*, *Csf1r*, *Cx3cr1*, *Fcrls*, *Hexb* (microglia) were used to determine purity. Transcripts per million (TPM) for each sample was determined from RNAseq data, and percentage of each cell type marker was calculated normalized to the total TPM of purity analysis genes within each sample.

#### JMP hierarchical clustering and principal components analysis

JMP Pro 10 statistical software (SAS) was used for hierarchical clustering, principal component analysis (PCA), and pairwise correlation analyses. For hierarchical clustering, gene expression data were normalized and clustered using k-means centroid-based clustering. PC analysis was performed on the entire gene expression datasets for each group, to show how the PCs explain the variation in the data. The PC points were derived from the eigenvector linear combination of the variables.

#### Gene ontology (GO) biological functions and pathway analysis

To determine the biological meaning of large gene lists, we used the Database for Annotation, Visualization, and Integrated Discovery (DAVID, version 6.8) [71] to calculate enrichment for gene ontology terms (GO Direct_BP; biological process). Gene lists were analyzed for overrepresentation of biological functions with Bonferroni, Benjamini, and FDR correction for multiple comparisons and in some cases functions with their respective *p* values of enrichment were graphed in Prism 6.0 (GraphPad). For Fig. 3a, genes from the AM module were sorted by expression ratio between MIA and Saline. Those genes with an expression ratio <1 in MIA versus Saline were sorted for expression ratio in MIA versus MIA + MG-REP. If direction of change was the same in MIA versus Saline and MIA versus MIA + MG-REP, the gene was included in the final list. The final lists of genes that is decreased in MIA versus Saline, and then increased in MIA + MG-REP versus MIA (or vice-versa) was input to DAVID and the top ten GO terms (biological process) were graphed. For all DAVID analyses, we used the default search settings, and a background list of 14,225 microglial expressed genes. To verify our GO findings, we performed 10,000-fold permutation tests to further establish the significance of GO overrepresentation within our overlapping gene lists. Raw and FDR-corrected (method: Bonferroni) *p* values were calculated.

#### Human gene expression data comparisons

Human orthologs of *Mus musculus* genes were determined using Biomart (http://useast.ensembl.org/biomart). Venn diagrams were created using Venny 2.1. Microglia gene lists were compared with published lists of ASD, schizophrenia, and intellectual disability-associated genes, synaptic and postsynaptic density molecules and FMRP interacting partners (see Supplementary Table 2 for gene lists and descriptions) using a hypergeometric distribution test (also known as one-tailed Fisher’s exact test) in Excel (Microsoft), using a background gene set of 13,757 genes with one-to-one mouse human orthologs, except for the Postsynaptic Density and Synaptic Protein lists, which used a background of 12,622 total protein-coding mouse-human orthologs (from microglial lists) and proteins with mouse orthologous (from Postsynaptic Density and Synaptic Protein lists). Before overlap analysis, human gene or protein lists were filtered for genes/proteins with mouse orthologs (5628 unique genes from all lists combined), and mouse microglia module genes were filtered for genes with human orthologs (12,487 unique genes). The background, excepting protein lists described above, was the sum of unique genes in these two lists (13,757) and included both mRNAs and ncRNAs. For mouse gene comparisons between differentially expressed genes and microglia module genes, the background was 13,483 unique mouse genes from the filtered dataset. To create microglia module-overlap analysis with human synaptic, schizophrenia- and ASD-associated gene modules (Fig. 2f), hypergeometric distribution was used to compare gene lists from microglial modules (IM, JM, MIA-IM, AM, and REP-AM) with publicly available lists of genes enriched in ASD brains, and genes associated with schizophrenia or synaptic interactions.

Genome-wide autism gene network predictions were made using ASD@Princeton [72], which uses a machine learning classifier that learns connectivity patterns of known ASD genes in a brain-specific network, and then uses these data-driven signals specific to ASD-associated genes to predict the level of potential ASD association for every gene in the genome, thereby providing predictions of ASD-associated genes, including candidate genes that have minimal or no prior genetic evidence. To create networks with ASD associated genes and AM (Fig. 2g), genes overlapping with the AM module and ASD modules M13, M16, and M17 were analyzed for gene ontology (GO) biological function enrichment (www.geneontology.org). The genes from the top two most significant functions by *z* score and *p* value (Bonferroni correction) were input to ASD@Princeton. To create networks of AM, human ASD module and ASD-predicted genes, the minimum biological relationship confidence was set to 0.3 (on a scale of 0.1 to 1) and the top 20 associated molecules were included in the network.

### Whole cell patch clamp recording

#### Preparation of brain slices for recording and filling

A total of at least three mice per group were used for electrophysiological recordings, and at least ten cells were recorded per animal. No formal statistical testing was used to pre-determine cell recording or imaging sample size, but we used sufficient sample size to ensure adequate power based on our previous published studies [52] and those generally employed in the field. Mice were fully anesthetized with isoflurane before decapitation with guillotine. The brain was immediately removed and placed into ice-cold oxygenated Ringer’s solution (concentrations, in mM: 26 NaHCO_3_, 124 NaCl, 2 KCl, 3 KH_2_PO_4_, 10 Glucose, 2.5 CaCl, 1.3 MgCl_2_; pH 7.4, chemicals from Fluka). The frontal cortex was mounted on an agar gel slab and sliced into 300 μm thick sections using a vibrating microtome in oxygenated ice-cold Ringer’s solution. The slices were transferred to room temperature (RT) Ringer’s solution for at least an hour before use in patch clamp experiments.

Slices were transferred to a submersion-type recording chamber affixed to the stage of an IR-DIC Microscope (Micro Video Instruments) and were consistently superfused in oxygenated RT Ringer’s solution throughout the patch clamp experiments. Slices were visualized under IR-DIC optics using an Andor Xyla digital camera. Layer V pyramidal neurons were identified by their distance from the pial surface of the cortex (400–700 μm). Electrodes were created from borosilicate glass with a Flaming and Brown micropipette puller (Model P-87, Sutter Instruments). Pipettes were filled with a potassium methanesulfonate internal solution (concentrations, in mM): 100 potassium methanesulfonate, 15 KCl, 3 MgCl_2_, 5 EGTA, 10 Na-HEPES, 1% biocytin (1% n-biotinyl-L-lysine), and had a resistance of 4–7 MΩ in external Ringer’s solution. Standard whole cell patch clamp recording techniques were used to examine the electrophysiological properties of the cells.

#### Physiological inclusion criteria

Neurons were recorded from prefrontal cortical areas including the prelimbic area, anterior cingulate, and dorsal premotor areas, by experimenters blind to the treatment groups. Cells were visually identified as layer V pyramidal neurons and were required to have stable access, low noise, and a resting membrane potential below −55 mV for inclusion in the study. Somata were located 400–700 μm from the pial surface. Layer V pyramidal neurons are both physiologically and structurally heterogeneous, and thus fall into one of several sub-types [73]. We used pre-established criteria to classify cells as RS or IB types: the first interspike interval (ISI) of the first voltage step to induce a spike train was compared with the subsequent ISIs in the train. RS neurons had an equal first ISI compared with second ISI. IB neurons had a shorter first ISI compared with subsequent ISIs [74]. A small number of rhythmic oscillating bursting neurons were also observed and excluded from this study. Significant differences were observed in the physiological properties of RS and IB neurons. Neuronal dendritic spine analyses were also performed only in IB cells.

#### Physiological analysis

PatchMaster acquisition software and EPC-9 or EPC-10 patch-clamp amplifiers (HEKA Elektronik) were used to acquire electrophysiological data including passive membrane, single action potential, and repetitive firing properties. A total of 37 physiological characteristics were assessed by experimenters blind to the treatment groups. FitMaster Analysis Software (HEKA Elektronik) was used to analyze passive and active membrane properties. The passive membrane properties, resting membrane potential (Vr), input resistance (Rn), and the membrane time constants (Tau) were recorded under current clamp conditions. Vr was measured as the voltage present when the current injection was zero. Rn and Tau were measured by injecting 20 mV steps starting at −160mV and ending at + 100mV. Tau was defined by fitting the membrane response to a small current injection (−20 or −40mV) to a single exponential function. Rn was assessed as the slope of a best-fit line through the *V–I* plot. Single spike properties [threshold, amplitude (amp), rise, decay, and duration at ½ amplitude (Dur ½)] were measured from the first single spike recorded on the 200 ms current step. Threshold was measured at the point the action potential’s slope was greater than 1 mV/ms. The amplitude was the maximum voltage recorded at the peak of the spike. Single spike rise was the time, in ms, for the action potential to rise from threshold to peak amplitude. Fall time was the time for the action potential to return to threshold from the peak amplitude. Dur ½ is the duration of the spike from half amplitude on the rise to half amplitude on the fall of the action potential. Repetitive firing of APs was determined as the number of spikes evoked at each step in a series of current steps that increased by 20mV from −100 to +120mV. sEPSCs were recorded for 2 min at a holding potential of −80 mV. sIPSCs were recorded at a holding potential of −40 mV. Following acquisition of spontaneous synaptic data, tetrodotoxin (1 μM) was added to the Ringer’s perfusion medium and, following a 5 min equilibration period, action potential independent mEPSCs were recorded as above. Synaptic data was assessed using MiniAnalysis software (Synaptosoft), with event detection threshold set at maximum RMS noise level (5pA). The sEPSCs and sIPSCs were automatically detected and manually confirmed to yield frequency and mean amplitude data. Averaged waveforms were created from all the EPSCs or IPSCs acquired for each neuron. From this, we calculated average EPSC and IPSC rise and decay time constants, and area under the curve (pA/ms).

### Immunohistochemistry

#### Slice processing of biocytin-filled neurons

Slices were fixed between filter paper in 4% PFA in 0.1 M PBS at pH 7.4 immediately after recording. Slices were stored for 2 d at 4 °C, washed in PBS, incubated in 1% Triton X-100/PBS for 2 h at RT, then incubated in a streptavidin-Alexa 488 (1:500; ThermoFisher) at 4 °C for 2 d. For long-term storage, slices were placed in PBS with sodium azide (0.005% NaN_3_ in 0.1 M PBS) until processing by immunohistochemistry.

#### Immunohistochemistry with microglial markers

To visualize microglia, we used a novel combination of immunostaining for IBA1, a cytoplasmic marker, and P2RY12, a cell-surface purinergic receptor specific to microglia in the brain. Immunohistochemistry was performed on 300 μm brain sections used for electrophysiology, based on a previously published protocol [75]. Briefly, 300μm sections were processed by antigen retrieval with 10 mM sodium citrate at 75 °C. The following steps were performed at 4 °C unless otherwise noted. Sections were incubated in 50 mM glycine in PBS for 30 min, then blocked in 5% BSA, 5% normal goat serum (NGS), and 0.2% Triton X-100 for 2 h. Sections were then incubated in primary antibodies (rabbit anti-mouse IBA1, 1:250, Wako, 019–19741 and polyclonal P2RY12 rabbit anti-mouse 1:2500, generated by Butovsky lab), in acetylated BSA (BSAc, Aurion) buffer (final concentrations: 0.1 M Na Phosphate buffer, 0.2% BSAc, 1% NGS and 0.1% Triton X-100) for 6 nights, with microwaving (in PELCO Biowave Pro Microwave Tissue Processor, Ted Pella) on d 1 (2 × 10 min) and 3 (1 × 10 min) of primary incubation, at 150 watts, 40 °C. Sections were then washed and incubated in secondary antibody (AlexaFluor546 goat anti-rabbit secondary antibody, 1:250, A-11010) in BSAc buffer for 2 nights, and microwaved at the same settings 1 × 10 min on day 1 and day 2. They were stained with Dapi for 30 min, washed overnight in 1x PBS, and mounted on slides with Prolong Gold antifade solution (Life Technologies, P36930) and number zero coverslips (Electron microscopy, 72198–10). Sections were cured at RT for at least 2 weeks prior to imaging.

### Confocal imaging, reconstruction, and analysis

#### Confocal imaging

For cell inclusion in morphological analyses, the criteria were pre-determined and were as follows: an intact soma, completely filled dendritic arbors, and no cut dendrites in the proximal third of the apical dendritic arbor. All confocal imaging was performed on a Zeiss LSM710 and acquired using Zen 2010 software (Zeiss). For imaging the entire neurons to assess whole-cell dendritic topology, stacks were acquired using a 40 × /1.3 NA oil-immersion objective (Plan-Achroma, Zeiss) at a resolution of 0.2 × 0.2 × 0.5 μm per voxel. For determination of spine subtype distributions and measurement of interactions with microglia, a second series of image stacks were acquired using the same 40x objective, at a resolution of 0.04 × 0.04 × 0.3 μm per voxel, from at least one complete basilar dendritic branch (~100 μm in length). To reduce signal blurring in the *z*-plane, each acquired stack of images was deconvolved using AutoQuant software (Media Cybernetics). Adjacent deconvolved image stacks were imported into Neurolucida (MBF Bioscience), aligned in 3D, and integrated into a single volumetric dataset. For analysis of complete neuronal structure, neurons were manually traced and reconstructed in Neurolucida. Scholl analyses were performed using Neurolucida.

#### Spine density/subtype and microglia interaction analyses

Spine subtype and microglia-neuron interactions were assessed from deconvolved and aligned 3D images in Neurolucida. Spine subtypes were assigned based on published methods [76]. Spines were classified into four subtypes: thin (head and neck <3 μm), stubby (no neck), mushroom (neck with head ≥0.6 μm) or filopodia (neck >3 μm in length). Microglia-spine and dendrite interactions were quantified from the same datasets and classified as follows: encapsulation (within 0.3 μm; one z-plane, and microglia is surrounding at least ½ the spine head diameter), apposition (within 0.9 μm; three *z*-planes, and microglia is surrounding less than ½ the spine head diameter), and proximity (within 1.5 μm; five *z*-planes) to microglia (see diagram in Supplementary Fig. 5g). Distances were measured from the most saturated three pixels of the neuron and microglia within the same optical slice, which allows 500-nm resolution at the ×40 oil-immersion objective’s NA 1.3. In order to calculate distances in 3D space, the Pythagorean theorem was used, modeling distances between neurons and microglia in the x, y, and *z* planes as equal to three sides of a right triangle.

#### Microglia morphology tracing

Microglial structures were manually traced by experimenters blind to the treatment groups using 8-bit confocal image stacks in NeuronStudio (CNIC, Mount Sinai School of Medicine, http://research.mssm.edu/cnic/tools-ns.html). One red marker was placed and resized to indicate the soma, and green, yellow, and purple markers were placed along the projections to mark the processes, branching points, and branch endings, respectively. The dimensions of all markers were appropriately resized to approximate the changing girth of the processes. Both maximum projection of the image stacks and a slice-by-slice view were used to ensure the continuity of microglial branches. Upon completion, sholl analysis, branch order analysis quantification, and convex hull volume analyses were conducted. Branch order analysis was performed as described in [77]. Centrifugal branch order analysis was used, where first order branches are those directly adjoining the soma, and branch order increases by one at each branch point as the microglia processes transverse outward. Convex hull volume analysis. The latter was run using the Trees toolbox in MATLAB (The MathWorks, Inc.) [78]. Measurements were quantified in an automated manner using a Rayburst sampling algorithm [58]. At least one microglial cell per basal dendrite analyzed was morphologically assessed.

#### Pre- and post-synaptic marker density and microglia interaction analyses

Brains were removed after perfusion fixation with 4% paraformaldehyde/PBS followed by post-fixation and cryoprotection with sucrose/PBS. They were cut coronally on a cryostat into 20 μm sections. Three sections from the frontal cortex area were used for immunohistochemistry and each section was separated by at least 200 μm. The sections were subjected to antigen retrieval with pepsin (Gene Tex, GTX28194) at 37°C, then blocked in 10% normal donkey serum, 1% BSA, and 0.1% tween in PBS. Sections were incubated with presynaptic marker-VGLUT2 (guinea pig, 1:250, Synaptic Systems, 135404), postsynaptic markerPSD95 (goat, 1:250, Abcam, ab12093), and mononuclear phagocyte markers (IBA1; Wako, 019–19741, and P2RY12 rabbit or rat polyclonal generated by Butovsky lab) diluted with 2% BSA, 0.025% tween in PBS for 24 h. Sections were then washed and incubated in secondary antibodies (AlexaFluor546 donkey anti-goat secondary antibody; 1:500, AlexaFluor647 donkey anti-guinea pig secondary antibody; 1:500, and AlexaFluor488 donkey anti-rabbit secondary antibody; 1:500) for 1 h at room temperature. All confocal microscopic imaging was performed on a Zeiss LSM710 and acquired using Zen2010 software (Zeiss). Serial *z*-stack images were acquired with n = 3 microglia per image and *z* = 0.5 μm. To reduce signal blurring in the *z*-plane, each acquired stack of images was deconvolved using AutoQuant software. For analysis, microglia were manually traced in the projection of the confocal serial zstack, and normalized to total surface area (μm^2^). Synaptic markers, which overlapped with microglia, were counted and normalized by microglia surface area. Counting of synaptic puncta was performed separately in each serial zstack using thresholding-based particle analysis and colocalization analyses in FIJI software.

#### Protein extraction and ELISA

Acutely isolated microglia (1–2 × 10^6^ cells) from E17 or P60 mouse brain were subjected to protein extraction in 300 μl of TENT buffer (25 mM Tris-Cl, pH 7.5, 100 mM NaCl, 2 mM EDTA, and 1% Triton X-100) with 1 mM phenylmethylsulfonyl fluoride (all from Sigma-Aldrich) by pipetting followed by vortexing for 10 s every 5 min for a total of 20 min on ice. The cell lysate was centrifuged at 20,000 × *g* for 30 min at 4 °C, and the supernatant was collected for determine protein concentration by BCA assay kit (Pierce/Thermo Fisher). 30 μg of protein was used per reaction. For the detection of NTRK2 (TrkB), PTN and NTN1, following commercial ELISA kits were used: Mouse TrkB ELISA kit (Boster Biological Technology, EK0849), Mouse PTN ELISA kit (Biomatik Corporation, EKU06717), Mouse NTN1 ELISA kit (LifeSpan Biosciences, LS-F5882). For the detection of NCAM2, CTNND2 and WNT5A, custom ELISA kits were developed according to the manufacturer’s instruction using anti-NCAM2 goat polyclonal antibody (0.3 μg/well Acris Antibodies GmbH, AP32136PU-N), biotinylated anti-NCAM2 goat polyclonal antibody using Antibody Biotinylation Kit (0.3 μg/ml, Pierce/Thermo Scientific, 90407), anti-CTNND2 mouse monoclonal antibody (0.3 μg/well, Santa Cruz Biotechnology, SC-81793, clone 40.1), biotinylated anti-CTNND2 rabbit antibody (1 μg/ml, Abcam, EPR17628), anti-WNT5A goat polyclonal antibody (0.3 μg/ well, R&D Systems, AF645), and biotinylated anti-WNT5A antibody using Antibody Biotinylation Kit (1 μg/ml, Pierce). For the standard molecules, we used CTNND2 protein (Abnova, H00001501-Q01), NCAM2 polypeptide (Everest Biotech, EBP06991) and WNT5A protein (R&D Systems, 645-WN-010). The ELISA method consists of capture and detection antibodies, streptavidin Poly-HRP (Pierce/Thermo Fisher, 21140), TMB solution (Thermo Fisher, N301), Stop solution (Thermo Fisher, N600) and Optical Density at 450 nm was read by a microplate reader (BioTek Instruments). The dynamic range of the detection of NCAM2, CTNND2, and WNT5A was 1–320 pg/ml according to the manufacturer’s instructions.

#### FACS-based purification of microglia

Briefly, mice were transcardially perfused with ice-cold Hanks’ Balanced Salt Solution and brains were dissected. Single cell suspensions were prepared and centrifuged over a 37%/70% discontinuous Percoll gradient (GE Healthcare), mononuclear cells were isolated from the interface. Cells were stained with the live/dead Blue kit (L34961, ThermoFisher) to remove dead cells. In order to distinguish resident microglia from recruited myeloid cells, we used an APC-conjugated rat monoclonal antibody that recognizes murine FCRLS (clone 4G11, custom development of Butovsky’s laboratory), which is expressed on microglia but not on infiltrating myeloid cells [79]. Isolated cells were stained with LY6C-PerCp-Cy5.5 (2 μg/ml, clone HK1.4, Biolegend), FCRLS-APC (3 μg/ml, rat clone 4G11) [80] and CD11B-PeCy7 (2 μg/ml, clone M1/70, BD Biosciences) antibodies to specifically sort resident microglia as LY6C^–^/FCRLS^+^/CD11B^+^ cells.

#### Confocal microscopic live-imaging of dendritic spine in neuron/microglia co-culture system

At DIV19, live imaging captured by confocal microscopy (LSM 880 with Airyscan, Zeiss) using ×63 objective at a resolution of 512 × 512 (8 bits/pixel). Z-stack images were captured every 2 min over 14 min imaging period per dendrite (*n* = 6 μm). Images were exported using Autoquant for spine analysis. Spine density was quantified at *t* = 0 and 14. The spine density was measured over a total dendritic length of ~50 μm using imageJ software (n = 6–9 dendrites from 4–5 neurons per group). Filopodia was defined as spine >3 μm as previously mentioned.

#### In situ hybridization and immunofluorescence of microglia

In vitro transcription of cRNA probes against the murine *Ncam2* 3′ UTR, murine *Wnt5a* 3′ UTR, murine *Ntn1* 3′ UTR, murine *Ctnnd2* 3′ UTR, and scramble under the control of the T7 promoter (taatacgactcactataggcg) were synthesized as double strand DNA fragment (See Supplementary Table 7). Digoxigenin (DIG)-labeled cRNA riboprobe was synthesized using DIG labeling mix (cat. no. 1 277 073, Roche Diagnostics GmbH, Mannheim, Germany) and Riboprobe In Vitro Transcription Systems (cat. no. P1420, Promega, Madison, WI) and purified using ProbeQuant G-50 micro columns (cat. no. GE28–9034, GE Healthcare, Boston, MA). Enhanced fluorescence in situ hybridization was conducted for the detection of each mRNA on brain tissue sections as described [14] using anti-DIG-POD Fab fragments (cat. no. 11 207 733 910, Roche, Branford, CT) and the TSA Plus Cy3 fluorescence system (cat. no. NEL745, Perkin Elmer, Waltham, MA). After the in situ hybridization, the sections were processed for immunofluorescence as described above to detect IBA1 antigen.

### Quantification and statistical analysis

To determine the relationships between different behavioral tests, spine density, spine-microglia interactions, electrophysiological measurements and microglial morphology, pairwise correlation analyses were performed in JMP Pro 10 (SAS) by calculating Spearman’s Rho, a correlation coefficient computed on the ranks of the data values. Color maps were created based on correlations and on *p* values of correlations. For all analyses including correlation analyses, alpha values for confidence intervals were 0.05. Data was normally distributed, although this was not formally tested for every dataset. Two-group comparisons were made using two-sided unpaired *t*-tests. Variances were tested by the Brown-Forsythe test. Four group comparisons were made using 2-way ANOVAs, with main factors being poly(I:C) (MIA) and MG-REP (treatment) or paired *t*-test within the group with Sidak method for *p* value adjustment. Data were corrected for multiple comparisons with Tukey’s post-hoc, unless otherwise stated in Figure legends. Six group comparisons for the social behavior analysis were made using 3-way ANOVAs, with main factors being poly(I:C) (MIA), MG-REP (treatment) and Chamber. For Figs. 1–5, outliers were removed using the ROUT test, with FDR = 1%, in Prism 8.0. For microglia morphology, data from one cell was considered an outlier if three or more values at individual branch orders were outliers by ROUT test of values within that branch order. *T*-tests and ANOVAs were performed using Prism 8.0 or R and correlations were performed in JMP Pro 10. Statistical analyses of RNA sequencing data were performed using R as described above.

### Data availability

RNA-sequencing and MG550 Nanostring data for this study will be deposited in the Gene Expression Omnibus with the primary accession codes (https://www.ncbi.nlm.nih.gov/geo/). The human ASD gene network data that supports the findings of this study are available at https://asd.princeton.edu [72]. All other data supporting the findings of this study are available within the paper and its Supplementary Data information files.

### Code availability

Code for the analyses described in this paper is available from the authors upon request.

## Supplementary Material

MIA Report Checklist

Suppl Fig 1

Suppl Fig 2

Suppl Fig 3

Suppl Fig 4

Suppl Fig 5

Suppl Fig legend

Suppl Table 1

Suppl Table 2

Suppl Table 3

Suppl Table 4

Suppl Table 5

Supple Table 6

Supple Table 7
