# Supplementary figures and images for: Inhibition of colony stimulating factor 1 receptor corrects maternal inflammation-induced microglial and synaptic dysfunction and behavioral abnormalities
Source: Mol Psychiatry. 2020 Feb 18;26(6):1808–31. doi: 10.1038/s41380-020-0671-2 (PMC7431382; doi:10.1038/s41380-020-0671-2)

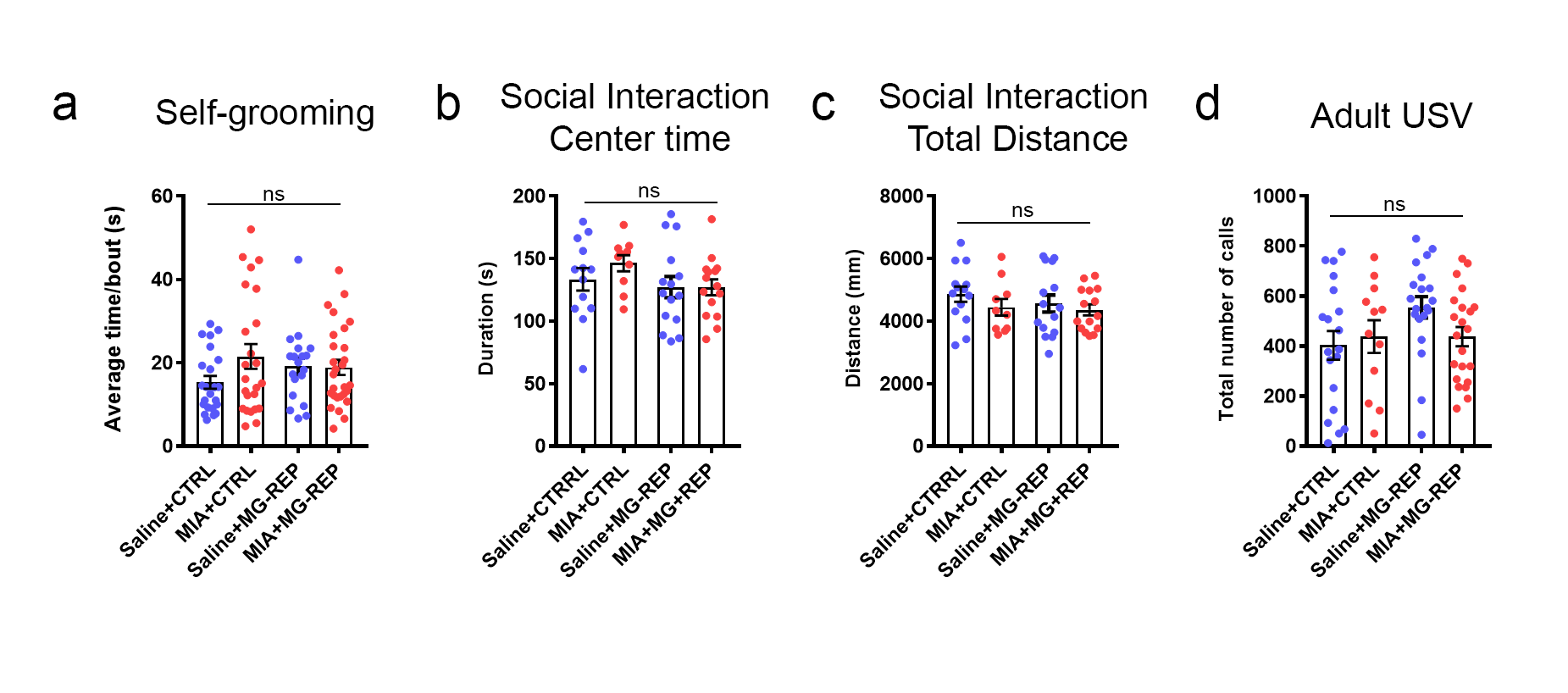

Supplement: Supplementary file 1 — Supplementary Figure 1 [file 41380_2020_671_MOESM1_ESM.tif]

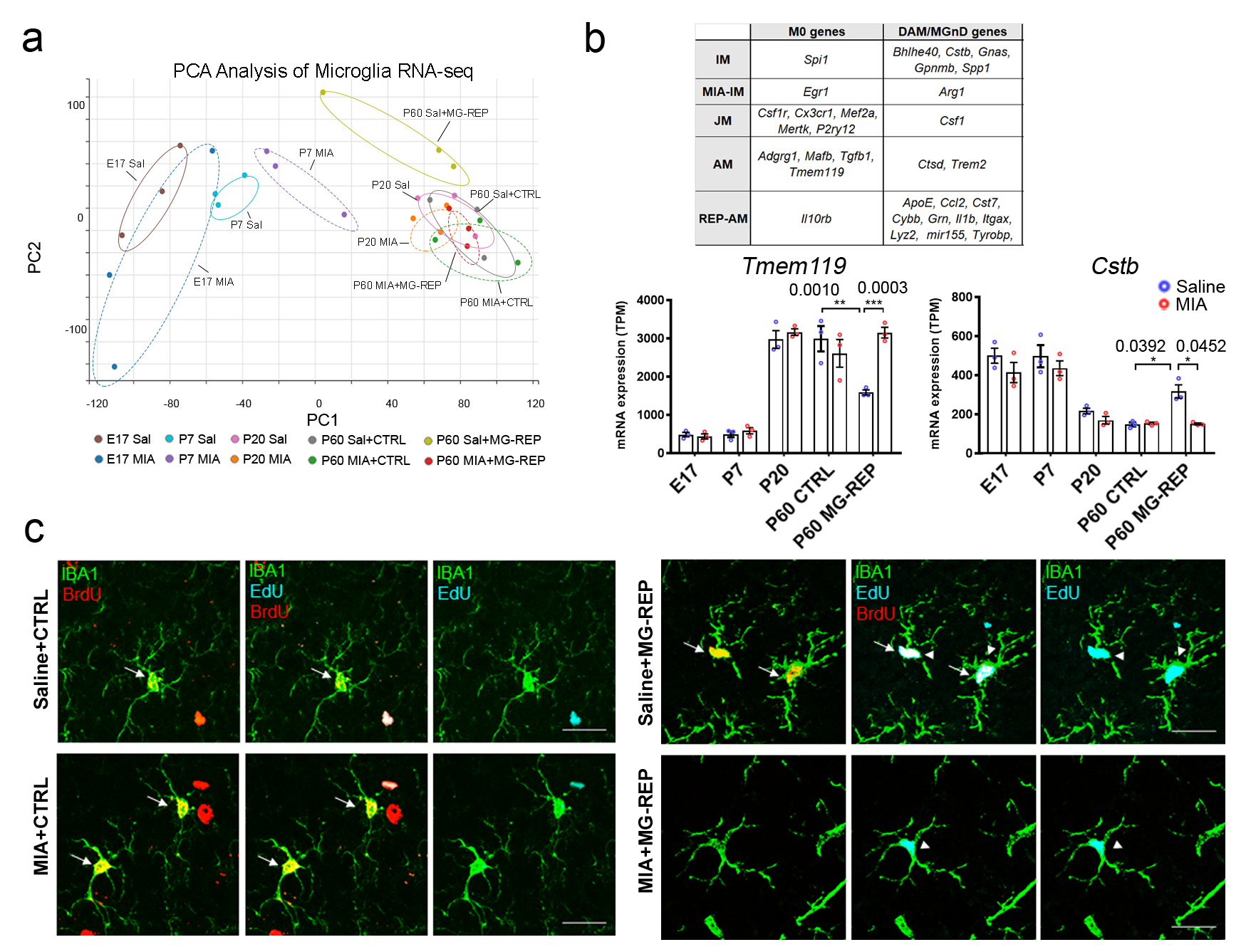

Supplement: Supplementary file 2 — Supplementary Figure 2 [file 41380_2020_671_MOESM2_ESM.tif]

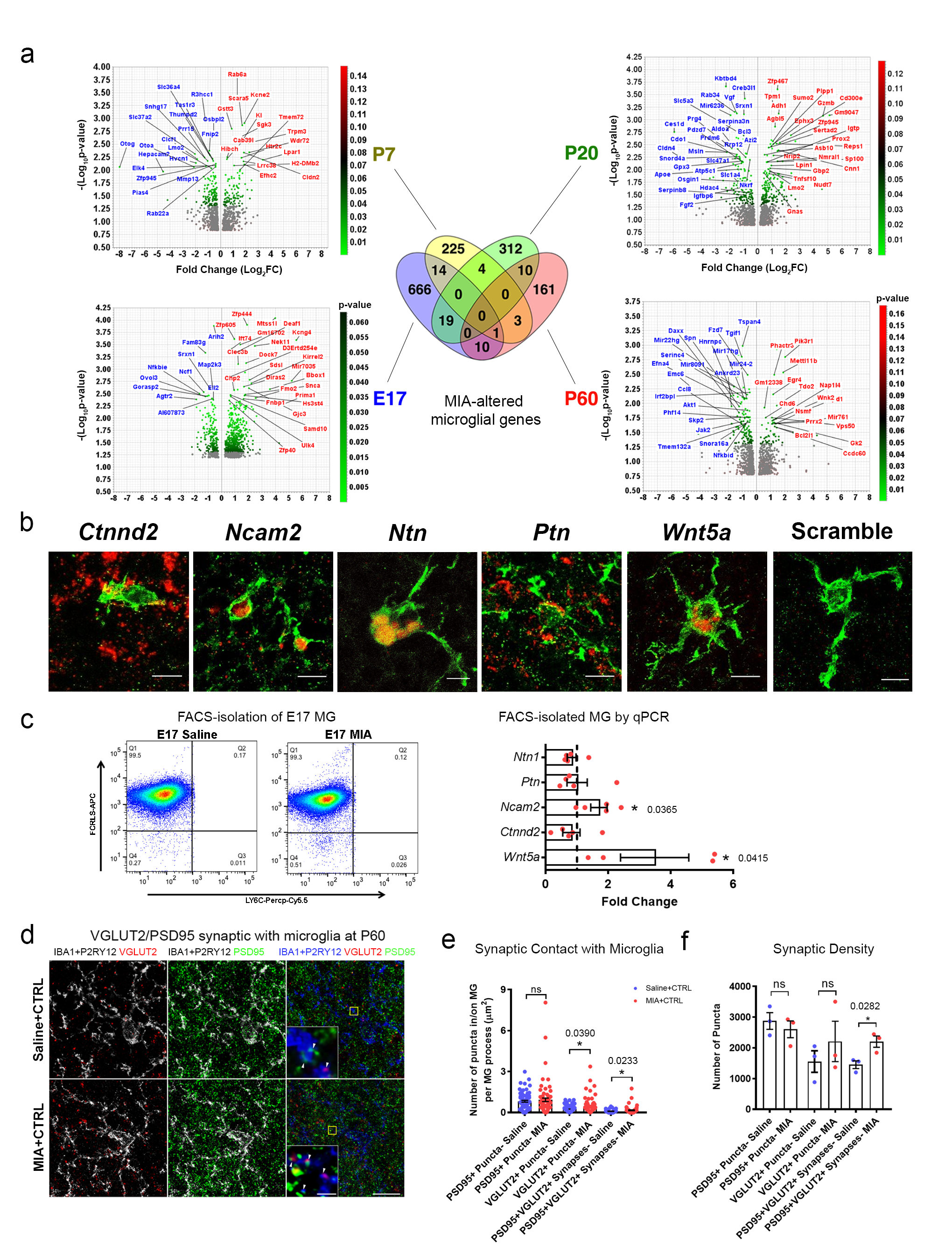

Supplement: Supplementary file 3 — Supplementary Figure 3 [file 41380_2020_671_MOESM3_ESM.tif]

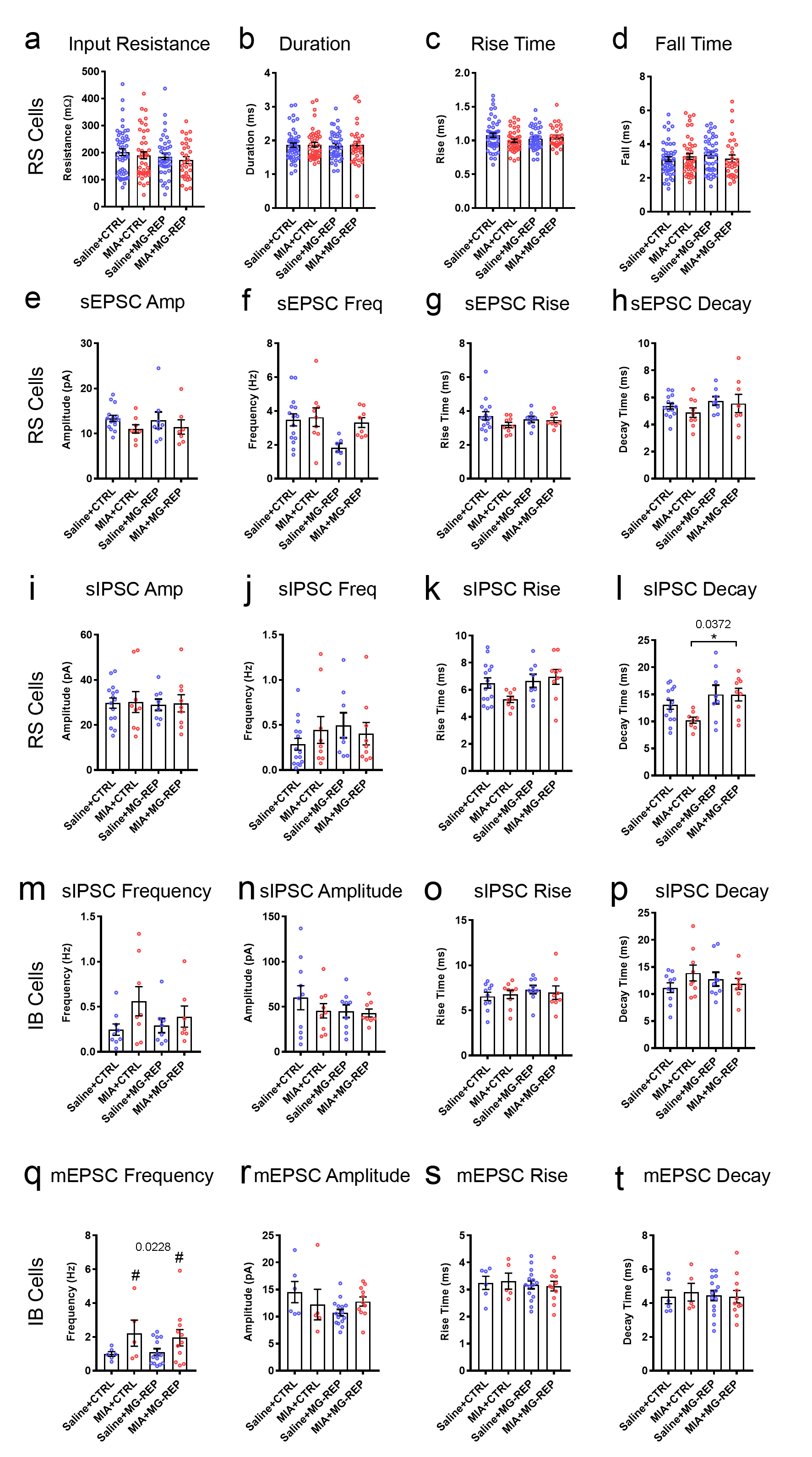

Supplement: Supplementary file 4 — Supplementary Figure 4 [file 41380_2020_671_MOESM4_ESM.tif]

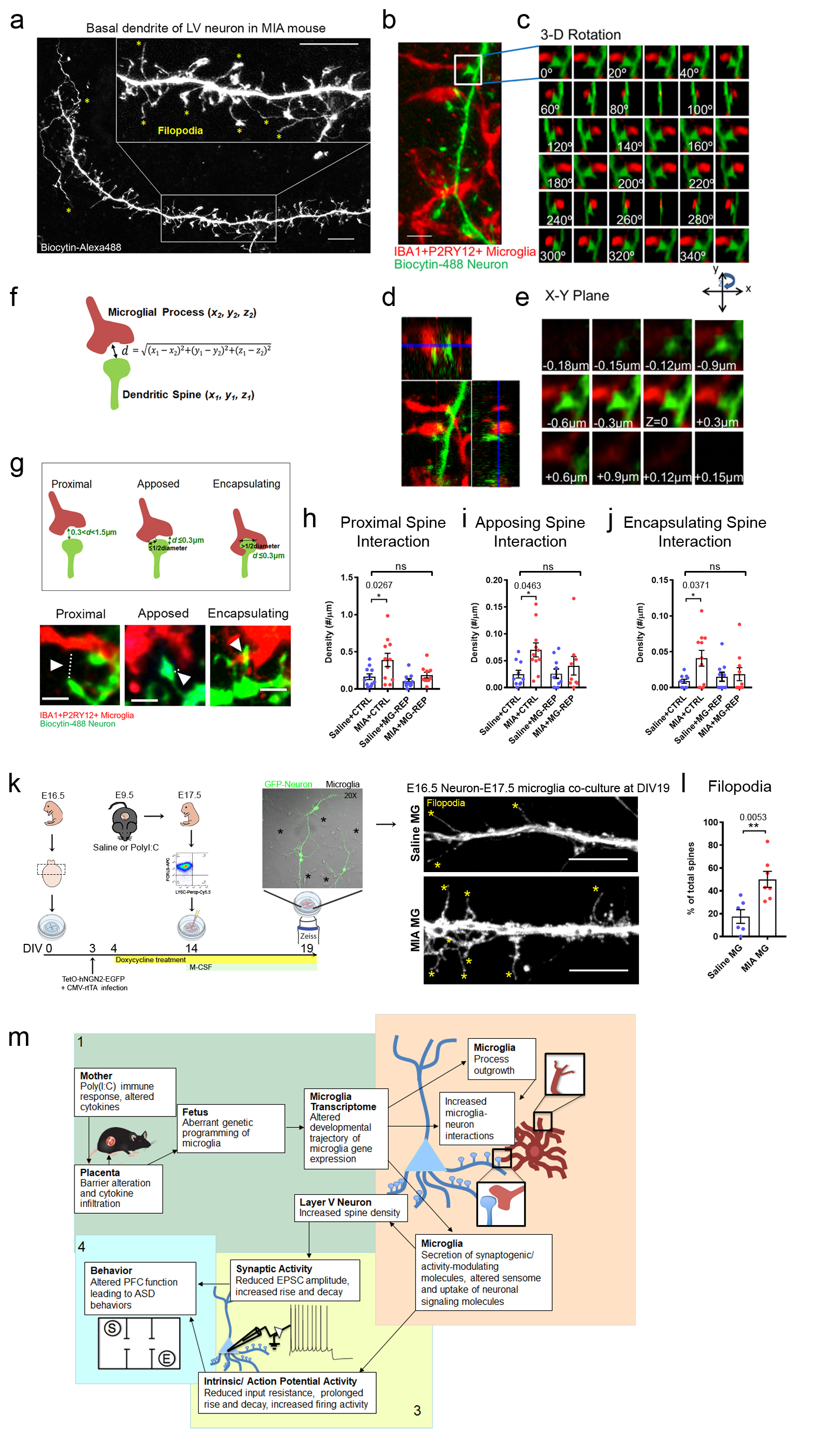

Supplement: Supplementary file 5 — Supplementary Figure 5 [file 41380_2020_671_MOESM5_ESM.tif]
