# Supplementary material for: Inhibition of colony stimulating factor 1 receptor corrects maternal inflammation-induced microglial and synaptic dysfunction and behavioral abnormalities
Source: Mol Psychiatry. 2020 Feb 18;26(6):1808–31. doi: 10.1038/s41380-020-0671-2 (PMC7431382; doi:10.1038/s41380-020-0671-2)
Supplement: Supplementary file 13 — MIA Report checklist [file 41380_2020_671_MOESM13_ESM.pdf]

Maternal Immune Activation Model Reporting Guidelines Checklist

ARRIVE Reporting Guideline & Recommendation	Arrive Item	MIA Model Specific Reporting Recommendation<br>***Please complete this chart for each point outlined below. If not applicable, write N/A***
**Study design**<br>➢Overview of immune activation issues<br><br>For each experiment, give brief details of the study design including:<br>a. The number of experimental and control groups.<br>b. Any steps taken to minimize the effects of subjective bias when allocating animals to treatment (e.g. randomization procedure) and when assessing results (e.g. if done, describe who was blinded and when).<br>c. The experimental unit (e.g. a single animal, group or cage of animals).<br><br>A time-line diagram or flow chart can be useful to illustrate how complex study designs were carried out.	6	MIA Specific Reporting:<br>a. General need for improved reporting in MIA model methods + reporting pilot data<br>○ Details on pilot data:<br>a) Control with Saline+CTRL, Saline+MG-REP, and experimental groups with MIA+CTRL and MIA+MG-REP<br>b) mice were randomly selected for injections, but due to increased spontaneous abortion rates and smaller litter size in PolyIC-injected group, MIA+CTRL and MIA+MG-REP were injected at a ratio of 1:1.5 to achieve similar offspring sample size. All experiments were performed and analyzed blinded/<br>c) all females were group housed prior to timed mating, but following timed mating, females were single housed after separation from male. All offsprings were group housed.
**Experimental procedures**<br>➢Compounds<br>➢Validation measures<br><br>For each experiment and each experimental group, including controls, provide precise details of all procedures carried out. For example:<br>a. How (e.g. drug formulation and dose, site and route of administration, anaesthesia and analgesia used [including monitoring], surgical procedure, method of euthanasia). Provide details of any specialist equipment used, including supplier(s).<br>b. When (e.g. time of day).<br>c. Where (e.g. home cage, laboratory, water maze).<br>d. Why (e.g. rationale for choice of specific anaesthetic, route of administration, drug dose used).	7	Provide details of:<br>a. Compounds – source, vehicle, preparation/storage, administration route, volume administered, whether anesthetics were used at time of immune challenge.<br>○ Name of compound: Polyinosinic-polycytidylic acid potassium salt<br>○ Catalogue number: sc-202767<br>○ Lot number: B0714,C1715, K2116, B2727<br>○ Vehicle control used: 0.9% Sodium Chloride USP – Preservative-Free, Dilue<br>○ Route of administration: IP<br>○ Volume administered: 150ul<br>○ Storage conditions: Stored as solid, and prepared fresh day of injection acco<br>○ Anesthetic (type, dose, duration) used:<br>None<br>b. Housing variables at injection - temperature of room at injection time, cage change at time of injection or not<br>○ Light cycle of animal housing room: 12hr/12hr<br>○ Time of day of injection: 12-2pm<br>○ Room temperature at injection time: 68-75F<br>○ Did a cage change occur at time of injection: NO

Kentner AC, Bilbo AD, Brown AS, Hsiao EY, McAllister AK, Meyer U, Pearce BD, Pletnikov MV, Yolken RH, Bauman MD. (2018). Maternal immune activation: reporting guidelines to improve the rigor, reproducibility, and transparency of the model. Neuropsychopharmacology, https://doi.org/10.1038/s41386-018-0185-7.

		c. Validation of immune activation – behavior, physiological indices and/or cytokine data, including pilot dosing data<br>○ Method used to verify immune activation:<br>in pilot dosing data, maternal blood plasma of IL-6 3-hrs post injection, cytokine qPCR of embryonic brain at E17 and P0, and offspring behavior were used to verify immune activation. In experimental data, behavior data was used to verify when<br><br>d. Validation of gestational timing – vaginal plug, estrous cycle, weight gain<br>○ Method of validating gestational timing:<br>Vaginal plug were used in combination with weight gain from E0 to E9.5. Females were combined with males, and vaginal plugs were checked 7-9am following morning.<br><br>Additional comments:<br>Injected temperature checked, and room temperature did not exceed 75 when injection.
**Experimental animals**<br>➢Species/strain/vendor<br><br>a. Provide details of the animals used, including species, strain, sex, developmental stage (e.g. mean or median age plus age range) and weight (e.g. mean or median weight plus weight range).<br>b. Provide further relevant information such as the source of animals, international strain nomenclature, genetic modification status (e.g. knock-out or transgenic), genotype, health/immune status, drug or test naïve, previous procedures, etc.	8	Provide details of:<br>a. Species – considerations for appropriate species (mouse, rat, non human primate, other)<br>○ Species: Mouse<br><br>b. Strain – variability in strain can influence model<br>○ Strain: C57BL/6J<br><br>c. Maternal/Offspring Physiological Variables at time of immune challenge – age, body weight<br>○ Maternal Age at challenge: 9-10 weeks<br>○ Maternal Body weight: 19-22g<br>○ Offspring Age at challenge: E9.5<br>○ Offspring Sex: Both males and females tested<br>○ Offspring Body weight: 9-12g at P21,22-26g at P60<br><br>d. Vendor – even within the same strain, vendor can influence endpoints<br>○ Vendor: JAX<br>○ Location of Vendor: USA<br>○ Room/area where animals originated from: AX8, Bar Harbor, Maine

		Additional Comments:
**Housing and husbandry**<br>➢Cage, ventilation, bedding, enrichment<br><br>Provide details of:<br>a. Housing (type of facility e.g. specific pathogen free [SPF]; type of cage or housing; bedding material; number of cage companions; tank shape and material etc. for fish).<br>b. Husbandry conditions (e.g. breeding program, light/dark cycle, temperature, quality of water etc for fish, type of food, access to food and water, environmental enrichment).<br>c. Welfare-related assessments and interventions that were carried out prior to, during, or after the experiment.	9	Provide details of:<br>a. Caging systems<br>○ *At breeding*<br>Material of cage: Polysulfone<br>Cage dimensions: 7.5 in x 5.625 in x 12.875 in<br>○ *After parturition*<br>Material of cage: Polysulfone<br>Cage dimensions: 7.620 in x 5.130 in x 15.010 in<br>○ *At weaning*<br>Material of cage: Polysulfone<br>Cage dimensions: 7.620 in x 5.130 in x 15.010 in<br><br>b. Animal Holding room<br>○ Temperature in room: 68-79F<br>○ Humidity in room: 30-70% Humidity<br>○ Ventilation system: Negative room pressure; Allentown IVC Hepa-filtered Su<br>○ Specific pathogen free [SPF]: NO<br>○ Are males & females housed in the same or separate rooms:<br>Housed in same room after weaning<br>c. Bedding exchanges/bedding type<br>○ Type of cage bedding used: Sani-chip bedding material<br>○ Frequency of cage changes per week<br>*during gestation:* 1<br>*during neonatal period:* 1<br>*following weaning:* 2<br><br>d. Breeding - bred on site or timed pregnant, how many different sires (are the same fathers breeding with both experimental and control dams)<br>Breeding location: On site in Boston Univeristy Medical Center barrier breeding facilit

- Gestational age at shipping: Timed pregnant mice E3-5 transported to
- Biological age of dams (if not listed in Section 8c):
- Number of Dams bred: 100 females
- How many times have dams been mated previously: 0-5 times
- How many times did the dams mate and not become pregnant: 0-5
- Are the dams primiparous or multiparous? All dams are primiparous
- What was the frequency of maternal handling during the gestational/neonatal period (e.g. cage cleanings, weighing, blood collection manipulations): Twice/week prior to injection for changing
- Biological age of sires: 10-15weeks
- Number of sires bred: 30 males
- How many times have sires been mated previously: 0-12 times
- How many times did the sires mate successfully (e.g. mating resulted in pregnancy, full term birth): n/a
- If bred previously, what was the interval between mating times:
- Are sires matched to experimental and control dams: NO
- Describe the mating design (1:1, 1:2 etc): 1M:2F

e. Social enrichment – number of cage companions
   - Number of cage companions prior to breeding: 5 females/cage
   - Gestational age when dam separated for parturition: E0.5 (separated
   - Number of cage companions at weaning: 2-4 males/cage; 2-5

f. Physical enrichment – describe enrichment devices, and when enrichment is in the cage (removed when pups born? Or present throughout study), does the enrichment type change? How frequently?
   - Describe what type of enrichment devices (and how many) are included in cage/housing room:

Two extra nestlets and 1 red plastic igloo added following separation at E0.5 until pups are born.

		○ Does enrichment type/access change across study? YES<br>○ If so, when does enrichment type/access change (e.g. enrichment removed prior to parturition and replaced in late neonatal period):<br>Following first cage clean at P4-5 following pups are born, red igloo is removed.<br><br>Additional Comments:<br>Maternal SFB results are negative in JAX.
**Sample size**<br>➢Litter versus offspring<br><br>a. Specify the total number of animals used in each experiment, and the number of animals in each experimental group.<br>b. Explain how the number of animals was arrived at. Provide details of any sample size calculation used.<br>c. Indicate the number of independent replications of each experiment, if relevant.	10	Provide details of:<br>a. Maternal N vs offspring N<br>○ What is the total number of dams/litters included in the study: 80<br>○ What is the total number of offspring per litter included the study:<br>2-5 males per litter for behavior, 1-5 males per litter for other analysis<br>b. Litter size and sex distribution<br>○ What size was each litter maintained at: Litters maintained at >3<br>○ What age did culling take place at: P21, litters sacd if <3 pups<br>○ How many males and females were maintained in each litter:<br>At least 2 males per litter until experimental<br>c. Cross fostering<br>○ Did cross fostering occur: NO<br>○ If so, at what age did cross fostering occur:<br><br>Additional Comments:

**Allocating animals to experimental groups**<br><br>a. Give full details of how animals were allocated to experimental groups, including randomization or matching if done.<br>b. Describe the order in which the animals in the different experimental groups were treated and assessed.	11	a. How many offspring per litter were used in each measure: 1-5 pups per litter<br><br>b. Randomization/Matching procedures<br>○ What procedures were used to assign animals to groups:<br>Based on the average litter size from C57blJ mice we randomly assigned 1:1.5 females to saline: polyIC condition to take into account the overall smaller liter size we have in PolyIC condition<br><br>c. Sex as a biological variable (behavioral and physiological outcomes)<br>○ Were both males and females evaluated in each behavioral and physiological outcome: NO<br><br>Additional Comments:
**Experimental outcomes**<br>➢Behavioral testing<br>➢Physiological endpoints<br><br>Clearly define the primary and secondary experimental outcomes assessed (e.g. cell death, molecular markers, behavioral changes).	12	a. Maternal behavior and pup interactions<br>○ If maternal care was evaluated, were there differences following immunogen challenge (if so, please briefly describe):<br>In pilot study, we individually separated juvenile mice from their mother and placed them into the center of a testing chamber con¬taining clean bedding ("novel nest") in one corner, and soiled bedding from their home cage ("home nest") in the opposite corner. MIA offspring displayed no olfactory deficit, as their home nest preference was intact. We then removed the pup, and placed an<br><br>b. Age(s) of offspring at behavioral testing/physiological evaluation endpoints:<br>P58-62 Self-grooming<br>P64-67 Social-interaction<br>P68 USV<br><br>c. Order of testing (e.g. behavioral test order)<br>○ Were animals evaluated in a counter-balanced order in terms of:<br>*presentation of tests to each animal:* NO<br>*order of experimental/control groups run through each test:* NO<br>○ What was the inter-test interval if a single animal underwent a battery of tests:<br>at least 4 days between behavior tests

		Additional Comments:<br><br>Experimenters are blinded so no counter-order of CTRL vs experimental groups.
**Statistical methods**<br><br>a. Provide details of the statistical methods used for each analysis.<br>b. Specify the unit of analysis for each dataset (e.g. single animal, group of animals, single neuron).<br>c. Describe any methods used to assess whether the data met the assumptions of the statistical approach.	13	a. Unit of analysis for each data set<br>○ Is the unit (n) of each analysis based on number of litters, or number of animals used per group:<br><br>Number of animals used for behavior, ISH, ELISA<br>Number of litter used for E17 qPCR and RNAseq<br>Number of cells used for electrophysiology, MG morphology<br>Number of dendrites used for spine analysis<br>Number of sections used for IHC assessing BrdU/EdU
**Other Disclosures**		Please make note of any other extraneous variables that you would like to report (e.g. fire alarms, construction, temporary relocations, other variables that you think we should be considering in our studies etc.):<br><br>Under high humidity or high temperature conditions, dams did not produce viable offspring due to spontaneous abortions.

The recommended use of this reporting form is to fill it out and include it as supplemental material for each of your laboratory's research publications. If there are difficulties utilizing/adapting this fillable form, please contact one of the corresponding authors to request a copy. The authors give permission for this table to be edited for use in reporting on other animal models (e.g. postnatal immune challenge models, early life stress models) as appropriate.

Kentner AC, Bilbo AD, Brown AS, Hsiao EY, McAllister AK, Meyer U, Pearce BD, Pletnikov MV, Yolken RH, Bauman MD. (2018). Maternal immune activation: reporting guidelines to improve the rigor, reproducibility, and transparency of the model. Neuropsychopharmacology, https://doi.org/10.1038/s41386-018-0185-7.
