# Supplementary material for: Inhibition of colony stimulating factor 1 receptor corrects maternal inflammation-induced microglial and synaptic dysfunction and behavioral abnormalities
Source: Mol Psychiatry. 2020 Feb 18;26(6):1808–31. doi: 10.1038/s41380-020-0671-2 (PMC7431382; doi:10.1038/s41380-020-0671-2)
Supplement: Supplementary file 14 — Supplementary Figures [file 41380_2020_671_MOESM14_ESM.pdf]

Supplementary Figure Legends

Supplementary Figure 1. Assessment of microglial depletion and repopulation effect on behavioral phenotypes in MIA offspring.

a, Average time per grooming bout. *n* = (23/9, 24/9, 19/11, 28/7) male mice/litters for (Saline+CTRL, MIA+CTRL, Saline+MG-REP, MIA+MG-REP). ns denotes no significance; determined by 2-way ANOVA (alpha = 0.05).

b-c, Time spent in the center of the three-chamber apparatus for social interaction tests (**b**); Total distance travelled in the three-chamber apparatus (**c**). No difference was observed between the saline or MIA ±MG-REP groups. *n* = (15/5, 15/9, 12/5, 15/4) male mice /litters for (Saline+CTRL, Saline+MG-REP, MIA+CTRL, MIA+MG-REP). ns denotes no significance; determined by 2-way ANOVA (alpha = 0.05). Graphs indicate mean ± s.e.m.

d, Communication deficits in male-female interactions was not observed in MIA mice in adulthood based on the number of ultrasonic vocalizations (USV) produced by male adult mice in presence of female mouse over 3 minutes. *n* = (19/6, 12/4, 20/5, 22/5) male mice/litters for (P60 Saline+CTRL, MIA+CTRL, Saline+MG-REP and MIA+MG-REP). Graphs show mean ± s.e.m.

Supplementary Figure 2. Microglia transcriptome analysis of genes altered across development and validation of gene expression of protrusion/neuritogenic molecules in murine microglia.

a, Principal component analysis (PCA) of RNA-seq profiles of acutely isolated microglia from E17 Saline (Sal), E17 MIA, P7 Sal, P7 MIA, P20 Sal, P20 MIA, P60 Sal+CTRL, P60 MIA+CTRL, P60 Sal+MG-REP and P60 MIA+MG-REP (n = 3 per group, 10 groups in total)

b, Representative M0 and DAM/MGnD genes differentially expressed in 5 microglial modules and expression levels of *P2ry12* and *Cstb* in different time points. * and ** denote $p < 0.05$ and 0.01 as determined by two-way ANOVA and Tukey's *posthoc*.

c, Representative images of BrdU (red), EdU (cyan), and IBA1 (green) microglia for Saline+CTRL (top), MIA+CTRL (bottom), Saline+MG-REP (top) and MIA+MG-REP (bottom). Arrow shows $BrdU^{+}IBA1^{+}$ original microglia. Arrowhead shows $EdU^{+}IBA1^{+}$ repopulated microglia. Scale Bar = 20 μm.

Supplementary Figure 3. Volcano plot analysis of differentially expressed genes in microglia across development and validation of gene expression of protrusion/neuritogenic molecules in microglia by *in situ hybridization*.

a, Venn diagram and volcano plots of significantly differentially expressed genes (DEG) in MIA versus Saline microglia at each age. Venn diagram shows number of genes with $p < 0.05$ between MIA and Saline microglia by exact t-test. Volcano plots: significantly differentially expressed genes between MIA+CTRL and Saline+CTRL. Red text: genes upregulated; blue text: genes downregulated by MIA. X-axis: log2 fold change in MIA+CTRL versus Saline+CTRL microglia, Y-axis: inverse log (p-value). Grey dots denote genes with $p \geq 0.05$.

The 14 common genes between E17 and P7 elements include *Scara5*, a ferrtin receptor that mediate non-transferrin-dependent delivery of iron. Since iron accumulation is reported in activated microglia, this suggests *Scara5* mediates iron accumulation in E17 and P7 microglia. *Irf9* mediates type I interferon signaling, which may represent sustained poly(I:C)-induced antiviral response in microglia during these periods. The 19 common genes between E17 and P20 include zinc finger transcriptional factors (*Zfp398, Zfp467*), iron transport (*Slc11a2*),

multiple drug and metal transport (*Slc47a1*), synaptic molecules (*Sv2c, Shisa6*) and exocytosis (*Clec3b*). This suggest active transport of iron and metals, exocytosis, and modulation of synaptic activities.

The 10 common genes between E17 and P60 include chemokine and TGFB signaling (*Ccl2, Tgif1*), acute phase reaction (*Orm3, Lpar2*), and inhibition of sodium-coupled chloride cotransporter (*Wnk2*). The only common gene among E17, P7 and P60 is *Ankrd53*, which is related to mitosis. There are 4 common genes between P7 and P20 and 3 common genes between P7 and P60. The numbers were too few to annotate significant cluster or functions. The 10 common genes between P20 and P60 include transcription factors (*Egr2, Prox2, Prrx2*), mTORC2 pathway (*Prr5*), cell surface sialoprotein (*Spn*), mitosis (*Ptp4a1*) and novel transmembrane proteins (*Tspan4, Tmem132a*). Further studies are necessary to understand the functional role of these molecules in microglia.

b, Representative images of the confocal microscopic images of the *in situ* hybridization (ISH) and immunofluorescence of E17.5 microglia. Pregnant mice were ip injected with saline or poly(I:C) at E9.5 and the embryos were perfused, frozen sectioned, and subjected to ISH at E17.5. ISH was performed for detection of mRNA of *Ptn, Ntn, Ctnnd2, Ncam2, Wnt5a,* and scramble control (red), followed by immunofluorescence of IBA1 (green). The sections were subjected to confocal microscopy imaging at 40x original magnification. Scale bar = 5 μm.

c, Validation of gene expression of neuritogenic molecules in murine microglia. Assessment of neuritogenic molecules in FACS-purified microglia from E17. Saline+CTRL and MIA+CTRL offspring. FCRLS^{+} LY6C^{-} microglial population (Q1) were 99.5% in Saline+CTRL group and 99.3% in MIA+CTRL group of gated CD11b^{+} cells. The changes in neuritogenic gene expression (*Ptn, Ntn, Ctnnd2, Ncam2* and *Wnt5a*) in E17 microglia were determined by qPCR

and shown as fold change in MIA+CTRL over Saline+CTRL group. N = 5 litters per group (total 25 pups for Saline+CTRL and 27 pups for MIA+CTRL groups). *p < 0.05 as determined by unpaired Student *t*-test. Graphs indicate mean ± s.e.m.

d, Enhanced microglial interaction with synapses in layer V mPFC neuropil by laser-scanning confocal microscopic analysis. Confocal images of presynaptic VGLUT2 (red), postsynaptic PSD95 (green) and microglial IBA1+P2RY12 (white in left and middle panels, blue in right panels) in the layer V neuropil of mPFC in Saline (top panels) and MIA mice (bottom panels). Microglia, IBA1+P2RY12, in maximum projection of a z-stack (z = 1.5 μm). Insets (right panels) show higher magnification of the images of synaptic interaction with microglia, white arrowheads show VGLUT2 or PSD95 co-localized with microglia (blue). 40× objective magnification, Scale bars = 10 μm (for insets: 1μm).

e-f, MIA increases microglial contact with synapses, indicated by co-localization of VGLUT2 and PSD95 with $IBA1^{+}P2RY12^{+}$ cells for Saline+CTRL and MIA+CTRL. **e**, Quantification of pre/post synaptic interaction with microglia (Number of $PSD95^{+}$ puncta, $VGLUT2^{+}$ puncta or $PSD95^{+}VGLUT2^{+}$ synapses co-localized with microglia). *n* = 81/3/3 microglia/ male mice / litters mice per group. **f**, Quantification of $PSD95^{+}$, $VGLUT2^{+}$ and $PSD95^{+}VGLUT2^{+}$ synaptic number analyzed. *n* = 3/3 male mice/ litters mice per group. $^{*}p < 0.05$ and $^{**}p < 0.01$, ns for no significance as determined by unpaired *t*-test.

Supplementary Figure 4. Intrinsic property, spontaneous inhibitory postsynaptic current (sIPSC) and miniature excitatory postsynaptic current (mEPSC) of layer V RS cells and sIPSC and mEPSC of layer V IB cells.

a-l, MIA has no effect on layer RS cells for **a-d**, their intrinsic properties, **e-h**, sEPSC and **i-l**, sIPSC except sIPSC decay, which is increased by MIA MG-REP group and Saline MG-REP group as compared to MIA CTRL group**. a-d**, *n* = (52/6/4, 45/6/4, 40/6/4, 33/7/6), **e-l**, (15/3/1, 9/3/1, 8/3/1, 8/3/2) cells/ male mice/ litters, for Saline+CTRL, MIA+CTRL, Saline+MG-REP, MIA+MG-REP.

m-p, MIA has no effect on spontaneous inhibitory postsynaptic current **(**sIPSC) properties of IB cells such as sIPSC frequency (M), amplitude (N), rise time (O) and decay time (P), *n* = 8-10 cells analyzed from 3 animals per group.

q-t, MIA increases miniature excitatory postsynaptic current **(**mEPSC) frequency (Q, MIA effect: $p = 0.0228$) that is not corrected via repopulation, but has no effect on other mEPSC properties of IB cells including amplitude (R), rise time (S), or decay time (T). *n* = (6/2/2, 5/3/3, 15/5/4, 11/4/3) cells/ male mice/ litters for Saline+CTRL, MIA+CTRL, Saline+MG-REP, MIA+MG-REP.

a-t, 2-way ANOVA: MIA effect $^{\#}p < 0.05$, Tukey's post-hoc: ns, no significance. Graphs indicate mean ± s.e.m.

Supplementary Figure 5. Confocal laser scanning microscopic imaging and quantification of microglial interaction with synapses in Layer V mPFC of P60 mice.

a, A representative image of a basal dendrite of a biocytin-filled Layer V pyramidal IB cells of P60 MIA+CTRL male mouse with multiple filopodia formation in basal dendrites (yellow asterisk). Scale bar = 7 μm.

b-e, Analysis of microglial interaction with dendritic spines. Representative 40× confocal images of microglia interaction with neurons: **b**, Basal dendrite of layer V pyramidal neuron (green) and

P2RY12/IBA1^{+} microglia (red). White box denotes area shown in **b**. **c**, Complete 3D rotation of spine-microglia interaction for quantification, at 10-degree increments, **d**, Detail of spine-microglia interactions around the white box inset in **b**, shown in *xy*, *yz*, and *xz* maximum projection images. **e**, Individual *xy* plane optical slices, imaged at z-increments of 0.3 μm.

f, Formula for the calculation of distance between the microglia process and dendritic spine. The distance shorter than 1.5 μm is defined as microglial interaction with dendritic spines.

g, Diagram (top) of microglia-spine interaction types and representative confocal images (bottom) of microglial processes (red) interacting with the dendritic spines of biocytin-filled layer V IB cells (green). Arrowheads indicate distance between the two processes.

h-j, Proximal (**h**), apposition (**i**) and encapsulating (**j**) spine-interactions normalized to basal dendrite length. *n* = (11/8/5/3, 11/8/5/4, 10/8/5/3, 10/4/3/3), dendrites/cells/male mice/litters for (Saline+CTRL, MIA+CTRL, Saline+MG-REP, MIA+MG-REP). $^{*}p < 0.05$, $^{**}p < 0.01$, $^{***}p < 0.001$ and $^{****}p < 0.0001$, ns denotes no significance; determined by 2-way ANOVA (alpha = 0.05) and Tukey's post-hoc. Graphs indicate mean ± s.e.m.

k-l, Confocal microscopic live imaging-assisted analysis of effect of MIA microglia on dendritic spine formation in murine primary cultured cortical neurons. **k,** GFP-labeled E16.5 primary cultured cortical murine neuron at DIV19 was co-cultured with FACS-purified E17.5 murine microglia from saline (Saline MG) or MIA offspring (MIA MG). n=6-7 dendrites per group from pooled E17.5 embryos/litter. Representative images show increased filopodia in neuronal dendrites co-cultured with MIA microglia **l,** The total % of filopodia spines is increased when co-cultured with MIA MG.

h-j and **l**. $^{*}p < 0.05$, $^{**}p < 0.01$, $^{***}p < 0.001$ and $^{****}p < 0.0001$, ns denotes no significance; determined by 2-way ANOVA (alpha = 0.05) and Tukey's post-hoc. Graphs indicate mean ± s.e.m.

m, Scheme of microglia mediation of MIA-induced neural deficits underlying ASD-like behavior.

1. Poly(I:C) injection stimulates immune response in mother, altering cytokines in the maternal-fetal placental barrier. Cytokine and sensome signaling molecules infiltrate the placental barrier, leading to altered microglial developmental gene expression trajectory in the fetus

2. Altered microglial transcriptome leads to aberrant microglial distal process outgrowth. Increased branching is associated with enhanced microglia-spine interactions in layer V basal dendrites, resulting in increased neuronal spine density.

3. Altered morphology and interactions of spines with microglia leads to, or alternatively is a result of, aberrant neurophysiological activity (intrinsic and synaptic).

4. Abnormal neuronal activity results in altered PFC function, underlying altered repetitive and social behaviors.
